# Disruption of Src Is Associated with Phenotypes Related to Williams-Beuren Syndrome and Altered Cellular Localization of TFII-I^1^,^2^

**DOI:** 10.1523/ENEURO.0016-14.2015

**Published:** 2015-03-30

**Authors:** Laleh Sinai, Evgueni A. Ivakine, Emily Lam, Marielle Deurloo, Joana Dida, Ralph A. Zirngibl

**Affiliations:** 1 Institute of Medical Science, University of Toronto, Toronto, Ontario, M5S 1A8, Canada; 2 Lunenfeld-Tanenbaum Research Institute, Mount Sinai Hospital, Toronto Ontario, M5S 3E1, Canada; 3 Programs in Genomics and Developmental Biology, The Hospital for Sick Children Research Institute, Peter Gilgan Center for Research and Learning, Toronto, Ontario, M5G 0A4, Canada; 4 Department of Physiology, University of Toronto, Toronto, Ontario, M5S 1A8; 5 Department of Psychology, Center for Biological Timing and Cognition, University of Toronto, Toronto, Ontario, M5S 3G3, Canada; 6 Department of Molecular Genetics, University of Toronto, Toronto, Ontario, M5S 1A8, Canada; 7 Lady Davis Institute, Jewish General Hospital, McGill University, Montreal, Quebec, H3T 1E2, Canada; 8 Department of Medicine, University of Toronto, Toronto, Ontario, M5S 1A8, Canada

**Keywords:** calcium channel, general transcription factor 2 I, mouse behavior, Src tyrosine kinase, Williams-Beuren syndrome

## Abstract

Src is a nonreceptor protein tyrosine kinase that is expressed widely throughout the central nervous system and is involved in diverse biological functions. Mice homozygous for a spontaneous mutation in *Src* (*Src* ^*thl/thl*^) exhibited hypersociability and hyperactivity along with impairments in visuospatial, amygdala-dependent, and motor learning as well as an increased startle response to loud tones. The phenotype of *Src* ^thl/thl^ mice showed significant overlap with Williams-Beuren syndrome (WBS), a disorder caused by the deletion of several genes, including General Transcription Factor 2-I (*GTF2I*). Src phosphorylation regulates the movement of GTF2I protein (TFII-I) between the nucleus, where it is a transcriptional activator, and the cytoplasm, where it regulates trafficking of transient receptor potential cation channel, subfamily C, member 3 (TRPC3) subunits to the plasma membrane. Here, we demonstrate altered cellular localization of both TFII-I and TRPC3 in the *Src* mutants, suggesting that disruption of Src can phenocopy behavioral phenotypes observed in WBS through its regulation of TFII-I.

## Abstract (precis)

Src tyrosine kinase phosphorylates the general transcription factor protein TFII-I, which is deleted in the neurodevelopmental disorder Williams-Beuren syndrome (WBS). We identified phenotypes such as increased sociability, visuospatial deficits, craniofacial abnormalities, and hyperactivity that overlap with symptoms of WBS in a mouse with disruption of *Src*.

## Significance Statement

Src tyrosine kinase phosphorylates the general transcription factor protein TFII-I, which is deleted in the neurodevelopmental disorder Williams-Beuren syndrome (WBS). We identified phenotypes such as increased sociability, visuospatial deficits, craniofacial abnormalities, and hyperactivity that overlap with symptoms of WBS in a mouse with disruption of *Src*. We also demonstrated altered cellular localization of TFII-I and of the TRPC3 calcium channel subunit, which is regulated by TFII-I. The finding that the *Src* null mouse phenocopies some features of WBS confirms its crucial role in the development and function of the nervous system, possibly through mediating changes in the cellular localization and function of TRPC3, and leading to altered agonist-induced calcium entry.

## Introduction

Src tyrosine kinases mediate a broad spectrum of physiological responses, including cell cycle control, proliferation, differentiation, migration, and survival, when activated in response to signals from diverse cellular receptors and extracellular stimuli (Thomas and Brugge, 1997). Src is highly expressed in the mammalian CNS and is involved in neuronal differentiation and neurite outgrowth (S.S. Kuo et al, 1997; W.L. Kuo et al, 1997; Hoffman-Kim et al, 2002). The NMDA receptor has been shown to be modulated by Src, and NMDA subunit phosphorylation and dephosphorylation is crucial for synaptic plasticity through the enhancement and suppression of NMDA receptor-mediated synaptic currents (Wang and Salter, 1994; Lu et al, 1998). The Src family kinases are also involved in platelet-derived growth factor and epidermal growth factor signaling (Kilkenny et al, 2003).

Src phosphorylates many downstream targets, one of which is General Transcription Factor 2-I (TFII-I), which undergoes phosphorylation-dependent shuttling between the nucleus and cytoplasm (Cheriyath et al, 2002). The GTF2I gene is commonly deleted in Williams-Beuren syndrome (WBS) (Merla et al, 2010), a neurodevelopmental disorder characterized by craniofacial dysmorphology, intellectual disability, deficits in visuospatial construction, relative strength in concrete language, social disinhibition, and nonsocial anxiety (Mervis and John, 2010). TFII-I has been described as a transcriptional regulator when in the nucleus (Roy et al, 1991), but acts as a regulator of agonist-induced calcium entry when in the cytoplasm through its competition with transient receptor potential cation channel, subfamily C, member 3 (TRPC3) for binding to phospholipase C-gamma (PLC-γ) (Caraveo et al, 2006).

The association of the Src pathway with a disorder involving altered social behavior suggests that Src itself may also be important for appropriate social interaction. Although *Src* knock-out mice have been generated previously, no behavioral assessments have been performed (Soriano et al, 1991; Kim et al, 2005). Here, we have characterized mice with a spontaneous null *Src* mutation for social and cognitive paradigms relevant to neurobehavioral syndromes such as WBS. We have identified increased social interaction and recognition with deficits in learning and memory, accompanied by alterations in downstream components of the Src pathway that are implicated in the etiology of WBS. Based on these findings, we propose that Src may be a connecting bridge between neurodevelopmental disorders with aberrant social behavior.

## Materials and Methods

### Identification of *Src* ^*thl/thl*^ mice

Affected mice were discovered during a routine observation of our animal colony. They were significantly smaller than their wild-type (WT) littermates and lacked incisors and some molar teeth. When maintained on a special mashed food diet, *Src^thl/thl^* mice survived until at least 1 year of age (end of the observation period). Intercross of heterozygous *Src* ^*thl/+*^ mice produced affected homozygous progeny at the expected Mendelian ratio.

**Figure 1.**
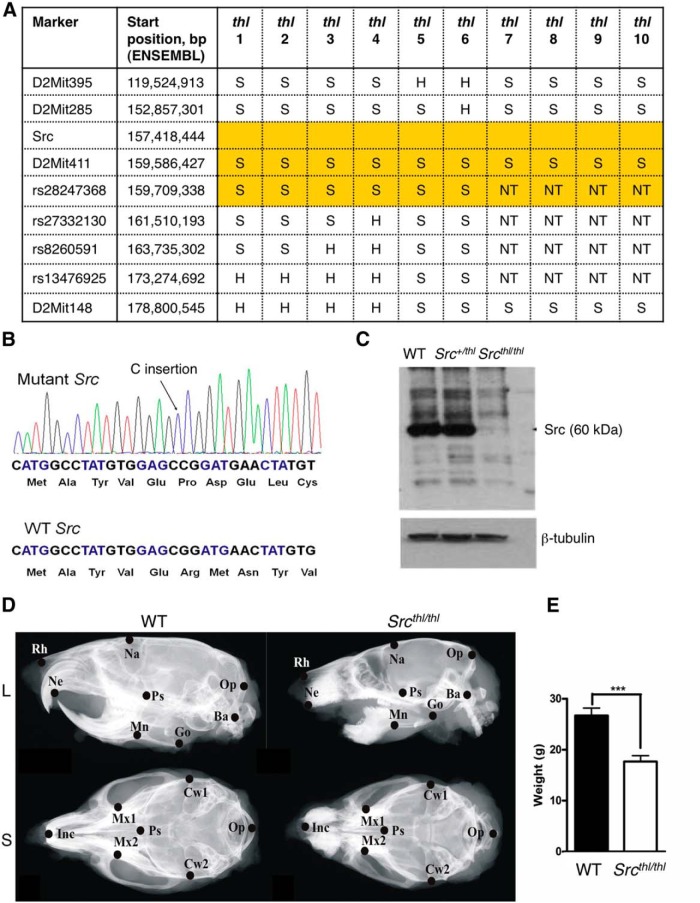
Craniofacial dysmorphology and growth retardation in *Src^thl/thl^* mice. ***A***, Whole genome scan in (S129 x C57BL/6) F2 toothless mice demonstrated linkage to D2Mit411. Additional markers were used to refine the critical interval (highlighted in yellow). S, Homozygous for S129 allele; H, heterozygous for S129 and C57BL6 alleles; NT, not tested. ***B***, Mutant mice have insertion of a C nucleotide in exon 12 of the Src gene. ***C***, No Src protein was detected in immunoblot of whole-brain lysates from *Src^thl/thl^* mice. ***D***, Faxitron images of WT and *Src^thl/thl^* mouse skulls in lateral (L) and superior (S) views with landmarks used for cephalometric analysis indicated and described in Materials and Methods. Abbreviations are defined in Table 1’s footnote. Analysis of the data is summarized in Table 1. ***E***, Weight of WT and *Src^thl/thl^* mice shown as mean ± SEM (*n* = 10 WT, *n* = 9 *Src^thl/thl^*).

Genome-wide mapping studies were performed on 20 affected mice using a panel of microsatellite markers positioned approximately 20 cM apart. For narrowing down the critical interval, both microsatellite and single nucleotide polymorphism markers were used. All markers and their corresponding positions, according to the Ensembl database, are listed in Figure 1*A*. Genotyping was performed at The Center for Applied Genomics, Toronto. After placing the Src gene within the critical interval, we sequenced the *Src* coding region from one affected mouse and confirmed the *Src* ^*thl*^ mutation.

All experiments were carried out with *Src^thl/thl^* and WT littermates. Animals were housed in groups of four or five per home cage and kept on a 12 h light/dark cycle, with food and water available *ad libitum*.

### Genotyping

Genotyping was performed by amplification of a 180 bp PCR product using the primer pair: 5′-CTATCCTTCTATCAGGAATAACCAG-3′ and 5′-GTTCTCCCCTACTAGGATATTG-3′, followed by digestion with the *Hpa* II restriction enzyme (Fermentas). PCR products from mice carrying the *thl* mutation gained a *Hpa* II restriction site that resulted in the 180 bp fragment being cleaved into four fragments (89 bp, 38 bp, 35 bp, and 18 bp) instead of three (107 bp, 38 bp, and 35 bp).

### Western blot analysis of Src

Protein lysates were prepared by homogenization of rapidly dissected whole adult brain in lysis buffer (10 mM TRIS-HCl 6.8, 2.5% SDS, 2 mM EDTA) containing protease and phosphatase inhibitor cocktails (Sigma-Aldrich). Lysates were adjusted to 1 mg/ml, and 50 μg of protein was used for SDS-PAGE (10% acrylamide gel) analysis and Western blotting. Western blots were probed with antibody directed against Src (Ab7950, Abcam). β-tubulin (Sigma-Aldrich) was used for loading control and protein signals were quantified using ImageJ (NIH, http://rsb.info.nih.gov/ij).

### Bone mineral density and craniofacial analysis

Nine-week-old male mice (four mice per genotype) were dissected to obtain the skull for faxitron analysis and the femurs and tibiae for bone mineral density determination by PIXImus densitometry (GE Medical Systems). Faxitron images were printed at the same magnification and the distances between established landmarks measured (Kiliardis et al, 1985; Lightfoot and German, 1998).

### Behavioral testing cohorts

Seven different cohorts of animals were used for behavioral testing. Where cohorts underwent two different kinds of testing procedure, there was a 2 week interval between the tests. The tests were as follows: Cohort 1, olfactory test then shock threshold; Cohort 2, open field then motor function; Cohort 3, elevated plus maze then fear conditioning; Cohort 4, sociability and social novelty then novel object recognition; Cohort 5, acoustic startle response then Morris water maze; Cohort 6, ultrasonic vocalization; Cohort 7 bone mineral density and craniofacial analysis. In Cohort 4, 2 weeks separated each social test (direct social interaction, three-chamber test, tube test).

### Sociability and social novelty recognition

#### Social approach and social novelty

We used the social approach task to assess the tendency to spend time with another mouse versus a novel object (an empty wire cage) and the ability to discriminate and choose between a familiar and new mouse. The apparatus consisted of a clear Plexiglas box (53 cm × 25.6 cm × 23 cm) divided into three chambers (outer chambers 19.5 cm in length; central chamber 13 cm in length). The outer chambers were divided from the central chamber by clear Plexiglas partitions (11 cm × 23 cm) containing an opening (7.3 cm × 7.3 cm). An empty wire cage (Galaxy Cup, Spectrum Diversified Designs) was used to hold the stranger mouse to prevent aggressive interactions and also support the availability of visual, auditory and olfactory cues. The wire cage was 11 cm in height, with a bottom diameter of 10.5 cm and bars spaced 1 cm apart. A beaker full of water was placed on the top of the cage to prevent it from moving. The cage was located in the center of each outer chamber throughout the experiment to permit social investigation.

The test was carried out according to previously published procedures (Nadler et al, 2004), on 8- to 10-week-old littermates with a habituation period followed by a 10 min experimental trial, and the unfamiliar mouse (Mouse 1) was alternated between right and left side chambers across subjects. Time spent in each chamber, number of entries, and time sniffing each wire cage was measured by an observer using Noldus software. In a subsequent 10 min trial, a new unfamiliar mouse was placed in the wire cage that had been empty during the previous session. The test mouse could choose between the first familiar mouse (Mouse 1) and the novel unfamiliar mouse (Mouse 2). Measures were scored as described above.

#### Direct social interaction test

The test mouse (male *Src^thl/thl^*) and social target mouse (weight- and age-matched unfamiliar male 129Sv/C57BL6/J) were placed in opposite corners of an unfamiliar neutral cage (30 cm × 17 cm × 12 cm), which was illuminated (280 lux) as previously described (File et al, 2001). Neutral cages were changed between experiments for each tested pair and each social target mouse was used only once. Scoring behavior parameters started with the first interaction and lasted for 5 min. During this time, social investigation and nonsocial behavior such as self-grooming, cage exploration (sniffing around the cage), and rearing were measured. The test mouse was not habituated to the unfamiliar cage and all measures were scored only after introduction of the social target mouse.

#### Tube test of social dominance

The tube is an additional measure of social behavior (Shahbazian et al, 2002; Spencer et al, 2005). In this test, 8- to 10-week-old WT and *Src^thl/thl^* littermates were placed in opposite ends of a clear acrylic cylindrical tube (3.5 cm diameter and 33 cm in length) and released at the same time. When they entered the tube, the mouse that backed out of the tube first was considered the loser.

#### Olfactory test

Olfactory function was measured by modifying the olfactory habituation/dishabituation task, as previously described (Wrenn et al., 2003). The mice were habituated to a novel clean testing cage for 5 min. Mice were then tested for time spent sniffing a Q-Tip suspended from the cage lid. The Q-Tip contained two different flavors for the habituation phase (almond extract; McCormick; 1:100 dilution) and dishabituation phase (lemon extract; McCormick; 1:100 dilution). A sequence of six trials for the almond extract assayed habituation to the same smell. Switching to the lemon smell on the Q-Tip assayed dishabituation (recognition that an odor is new). Each swab was presented for a 2 min period for six trials for the almond extract and one trial for the lemon extract. The time spent sniffing the Q-Tip was measured with a stopwatch by an observer. Sniffing was scored when the nose was within 2 cm of the Q-Tip.

### Ultrasonic vocalization

Ultrasonic vocalization (USV) recordings were carried out on mice between 11 and 16 weeks of age. In the male−female paradigm, mating-induced USVs and video recordings were obtained as follows. USVs were obtained with a D1000X ultrasound recorder (Pettersson Elektronik AB) for 5 min at a sampling frequency of 250 kHz. The microphone was suspended from a Plexiglas chamber put on top of the mating cage. Spectrographs (20-125 kHz) were generated by discrete Fourier transformation (256 bins) and analyzed with Avisoft SASLab Pro Software v4.39 (Avisoft Bioacoustics). A Vostro 1710 laptop running Windows XP was used for analysis of sonograms by a trained observer who was blind to genotype.

The female mouse was removed from the home cage and placed in a clean standard polyethylene cage (22 cm × 30 cm × 15 cm) with fresh bedding. The clean cage was brought into the testing room adjacent to the housing room, illuminated by a 40 watt red bulb. The top of the cage was removed and replaced with the Mating Chamber Addition, which consisted of four polyethylene extension walls with an open top and with an arm suspended in the center of the chamber. The microphone was suspended in the center of the cage, 12 cm above the floor. A video camera outside the lateral wall was positioned to record behaviors synchronously for further analysis. WT females were paired with either WT or *Src^thl/thl^* males. The female was left to habituate to the testing room for 5 min (Gourbal et al, 2004; Hoffman et al, 2012) to reduce stress and focus the attention of the female on the introduced male, thus increasing the probability of vocalization (Zampieri et al, 2014). The male was then introduced to the cage and pairing was allowed to continue for 5 min. Audio and video recordings were obtained for the entire duration of the pairing.

In our female−female social reunion paradigm, same-litter females were reunited following a period of separation. WT females were housed in separate home cages from *Src^thl/thl^* females due to competition for food, so female−female reunions were always between mice of the same genotype. The day prior to testing, one mouse was removed from the home cage and put into a clean standard polyethylene host cage. Twenty hours following separation, the host cage was taken to the testing room and the host was left to habituate for several minutes without food, water, or nesting to minimize distraction. The top of the cage was removed and replaced with the Mating Chamber Addition for the same purpose as in the male−female paradigm. Following habituation, a mouse from the home cage, “the visitor”, was introduced into the host cage and the reunion was allowed for 5 min. USVs and video recordings were obtained for the entire trial. Total USV counts generated each minute were counted and grouped into three categories [Type 1, flat; Type 2, broken; Type 3, frequency modulated, as previously described (Wang et al, 2008)] for the entire trial. The total number of calls and type of calls within each minute were scored.

### Exploratory activity and anxiety

Motor activity of 6-week-old mice was measured in the open field, and anxiety of 8-week-old mice was assessed in the elevated plus maze. Anxiety-like behaviors were measured as time spent in the center of the open field and as time spent in the open arms of an elevated plus maze according to previously published procedures (Kirshenbaum et al, 2011). Briefly, in the open field, mice were placed in the center of a transparent Plexiglas arena (41.25 cm × 41.25 cm × 31.25 cm) illuminated by 200 lux, and total distance travelled, time spent in the center (31 cm × 31 cm) and rearing were recorded for 30 min. The elevated plus maze consisted of a central platform (5 cm × 5 cm) with two opposing open arms (25 cm × 5 cm) and two opposing arms enclosed by opaque Plexiglas walls (25 cm × 5 cm × 30 cm). All arms were made of opaque Plexiglas and the entire apparatus was elevated 50 cm from the floor. Experiments were conducted in a dark room and open arms were brightly illuminated (700 lux). Mice were placed on the central platform facing a closed arm and the number of entries into each arm and duration in each arm was scored for 5 min. Time spent in the center of the maze was not recorded.

### Sensory and motor function

All tests of motor performance were carried out on 8- to 10-week-old littermates.

#### Rotarod

Mice were tested for two trials per day with a 60 min intertrial interval for 4 consecutive days. We used an Economex Rotarod apparatus (Columbus Instruments). The 3 cm ribbed plastic rotating axle was hung at a height of 30 cm above the base of the apparatus. Mice were placed on top of the rod, facing away from the experimenter. In this orientation, forward locomotion opposite to rotation of the rod is necessary to avoid falling. During the stationary habituation session, each mouse was first observed for 60 s without any rotation to allow the animals to become accustomed to the apparatus. The axle was then adjusted for a fixed constant motor speed of 5 rotations per minute (rpm) and each mouse observed for a total of 90 s. Next, beginning at 5 rpm, the rotation was accelerated in increments of 0.1 rpm/s and the latency to fall off the axle was recorded for each mouse for a maximum period of 5 min. Average time spent on the rotarod was calculated for each day.

#### Grip strength

The maximal muscle strength of the forelimbs, and both forelimbs and hindlimbs together was measured with an isometric transducer attached to a 3 mm diameter metal bar (Ugo Basile). For the forelimb measurements, each mouse gripped the bar with its forelimbs and was then slowly pulled backwards until it released the metal bar. The transducer measured the maximal grip strength in grams. Five trials were performed in each testing session, and the mean value was calculated. For the hindlimb measurements, a similar method was used but each mouse was allowed to grab with both its front and hind paws. The forelimb measurement was subtracted from the combined forelimb and hindlimb measurement to obtain the hindlimb grip strength.

#### Balance beam test

Mice were acclimated to a round, 30-cm-long, 3-mm-wide beam elevated 28 cm above a padded base. A 60 W lamp at the starting platform served as an aversive stimulus and the opposite end of the beam was in a darkened escape box on the arriving platform. Traversal time and number of slips were measured as mice travelled across the beam. All testing was performed in triplicate and mean values were used for subsequent statistical analyses.

### Acoustic startle response

The acoustic startle response test was carried out on 10-week-old littermates. Each mouse was placed into the startle chamber and allowed to acclimatize for 15 min. The mouse was then presented with 25 ms startle stimuli of varying intensities (70-120 dB), with an interstimulus interval of 25-30 s. Startle stimuli were presented in three blocks each composed of two presentations of the 11 stimulus intensities given in a pseudorandom order. The average startle amplitude for each stimulus intensity was calculated from the three blocks.

### Learning and memory tasks

#### Morris water maze

The Morris water maze (MWM) was used to test spatial memory. Prior to the test, mice were handled for 2 min per day for 7 consecutive days. Testing was carried out on mice between 11 and 16 weeks of age. Mice were given two trials per day, with a 30 s intertrial interval for 6 consecutive days. This challenging approach was used to enable us to detect even subtle deficits in visuopatial learning and memory. Two hours after the training on day 6, a probe trial was given to test spatial learning. Mice were placed in the pool at the opposite quadrant of the platform position and were given 60 s to search for the platform.

The MWM consisted of a white plastic 117-cm-diameter circular pool filled with water (with constant 26 °C temperature) made opaque by white latex paint. Around the outside of the pool, 2D and 3D visual cues were displayed in a dimly lit room. The pool was divided into four equal quadrants labelled northeast, southeast, southwest, and northwest. A hidden platform (10 cm diameter) submerged 1 cm below the water surface was placed at the center of one of the four quadrants (the target quadrant). A closed-circuit video camera was mounted directly above the center of the pool and connected to an image analyzer (HVS Image), which digitized the path data.

Mice were given two trials per day, with a 30 s intertrial interval for 6 consecutive days. During the visible platform trial, the platform remained submerged, but was flagged with a small spatial cue. The mouse was first placed on the platform for 30 s, then placed in the water at a pseudorandom start position. The mouse was given a maximum of 60 s to find the platform, following which it was placed back onto the platform. After 30 s on the platform, this training procedure was repeated once more. The platform remained at the same position during all trials. The hidden platform trial was performed in the same way as the visible platform trial, but with the visual cue removed. Two hours after the training on day 6, a probe trial was given to test spatial learning. Mice were placed in the pool at the opposite quadrant of the platform position and were then given 60 s to search for the platform. For the purpose of statistical analysis, crossings in the quadrant containing the target platform were compared to the averaged crossings of a similarly sized and positioned area within each of the three nontarget quadrants.

#### Fear conditioning

Fear conditioning was performed in a testing chamber (25 cm × 30 cm × 25 cm, MED Associates) fitted with a removable grid floor of 36 stainless steel rods (3.2 mm diameter, 4.7 mm apart) connected to a constant current shock generator, and an amplifier and speaker. A 12 inch, 8 W fluorescent tube (GE Lighting Canada) illuminated the chamber interior. A computer running automated fear conditioning software (FreezeFrame; Actimetrics Software) administered foot shocks and auditory tones. Video images were recorded from the chamber and the activity of subjects was recorded throughout the experiment. Immediately prior to training, the chamber was cleaned with 70% ethanol and a white cloth covered the front. Conditioning consisted of two pairings of an auditory tone with a continuous foot shock. Each mouse was placed inside the conditioning chamber for 2 min before the onset of a conditioned stimulus (CS; an 85 dB tone), which lasted for 30 s. A 2 s unconditioned stimulus (US; foot shock, 0.5 mA) was delivered immediately after the termination of the CS. Each mouse remained in the chamber for an additional 120 s, followed by another CS−US pairing. The mouse was returned to its home cage after another 2.5 min.

Approximately 24 h later, each subject was returned to the chamber and percentage of freezing was monitored for 3 min in the conditioned context. The activity of each subject was recorded at 0.25 s intervals using the FreezeFrame automated fear conditioning software (Actimetrics Software), which detects any kind of movement. The mean activity during the context exposure was calculated, the mean baseline activity subtracted, and the resulting figure used as a measure of contextual learning. Four hours later, the context was changed by covering the grid floor with a sheet of white PerspexTM (polymethyl methacrylate), inserting two sheets of transparent Perspex into the chamber to give it a prism shape, cleaning the chamber with 1% vinegar, covering the front door with a striped black and white card, and turning on the ceiling lights. Each mouse was placed into the altered chamber, and allowed 3 min for exploration in the novel environment, after which the auditory tone of 3 min duration was delivered. The mean activity during cue delivery was calculated, the mean activity in the novel context (prior to the presentation of the tone) subtracted, and the resulting figure taken as a measure of cue-associated learning.

#### Shock threshold

Shock threshold was assessed by delivering foot shocks starting at 0.075 mA and increasing by 0.05 mA every 30 s in the conditioning chamber. The experiment was terminated at a shock intensity sufficient to induce vocalization. Testing was carried out on mice between 11 and 16 weeks of age.

#### Visual-object and novelty recognition

The visuospatial and novelty recognition task was carried out as previously described (Ng et al, 2009). The task was performed in a transparent Plexiglas open field (41 cm × 41 cm × 31 cm) equipped with infrared beams to detect locomotor movements (model 7420/7430; Ugo Basile). Four of the objects used in this task were similar in shape, color, and material (approximately 7 cm × 6 cm × 6 cm) for the spatial recognition task, and one object was different for the novel object recognition task. Animal behavior was recorded by an observer and analyzed using The Observer 5.0 (Noldus). On the test day, each mouse was individually placed in the center of the empty arena to habituate for 5 min. The mouse was then placed in a holding cage for 2 min. Two objects were placed in specific positions in the corner of the arena and another two objects were positioned in the center of the arena. The mouse was returned to the center of the arena and allowed to explore the objects for three continuous 5 min sessions (training phase). Training to object exploration was measured by recording the time spent exploring the objects across the sessions. A mouse was considered to be exploring an object if its snout was in contact with the object. At the end of the training phase, the mouse was again placed in the holding cage for 2 min and the two center objects were moved to the corner positions in order to assess response to a spatial change.

The mouse was returned to the arena and the time spent exploring the displaced and nondisplaced objects was recorded for 5 min (spatial change phase). Reaction to a spatial change was assessed by calculating the ratio of the amount of time spent exploring the displaced object over the total time spent exploring the nondisplaced objects (preference index) by each mouse. Mice would be expected to spend more time exploring the displaced object than the nondisplaced objects due to the residual memory of the nondisplaced object and its position. Response to novelty was also examined. Directly after the spatial change phase, the test subject was returned to the holding cage for 2 min, during which one of the familiar nondisplaced objects in the arena was replaced by a novel object in the same location. The mouse was returned to the center of the arena for a 5 min period. Measurements were taken as described for the previous phase, and the response to novelty change was evaluated by calculating the ratio of the amount of time spent exploring the novel object over the total time spent exploring the familiar objects (preference index) by each mouse. Mice would be expected to spend more time exploring the novel object than the familiar object due to the residual memory of the familiar object. Testing was carried out on mice between 11 and 16 weeks of age.

### Cellular localization of TFII-I and TRPC3

#### Cellular fractionation

Adult mice were killed by cervical dislocation, and brains were removed immediately. One hemisphere of the brain was finely diced and homogenized with 2 ml homogenizing buffer (HB) containing 25 mM KCl, 1 mM MgCl_2_, 20 mM HEPES (pH 7.4), 1 mM EGTA, 0.2 mM DTT, 0.32 M sucrose, 1 mM PMSF, 1 mM protease inhibitor cocktail (P8340; Sigma-Aldrich), and 1 mM sodium orthovanadate. Samples were homogenized by five strokes of a Teflon homogenizer followed by an incubation of 2 min on ice in HB and then homogenized with 20 strokes of a Dounce homogenizer. All spins were performed at 4 °C. The homogenate was first spun at 600 *g* for 10 min. The pellet (P1) was further treated to obtain the nuclear fraction, and the supernatant (S1) was stored at −20 °C for later fractionation. Resuspended in 1 ml HB, P1 was passed through a 27 G syringe and spun again at 600 *g* for 10 min. The pellet was then resuspended in 1 ml nuclear buffer 2.2 M sucrose, 1 mM MgCl_2_, 10 mM HEPES, pH 7.4) and homogenized by eight strokes of the Teflon. After spinning the homogenate at 75,000 *g* for 75 min, the resulting nuclear pellet was resuspended in RIPA buffer (10 mM Tris-HCl, pH 8.0, 100 mM NaCl, 0.1% SDS, 0.5% sodium deoycholate, 1% NP-40, 1 mM EDTA, 1 mM protease inhibitor cocktail) and stored at −20 °C. S1 was spun at 10,000 *g* for 15 min, and the supernatant was further spun at 100,000 *g* for 60 min to obtain the cytosolic fraction (supernatant). The pellet was resuspended in 1 ml HB and spun again at 100,000 *g* for 60 min followed by a last wash in 0.5 ml HB at 100,000 *g* for 60 min. The pellet was then resuspended in RIPA buffer and constituted the membrane fraction.

##### Western blot analysis

Fractions were quantified by D_C_ Protein Assay (Bio-Rad), and 30-40 μg was used for Western blot analyses by SDS-PAGE on 8% acrylamide gels. After blotting onto a PVDF membrane, the nuclear fraction was probed with anti-TFII-I mouse monoclonal antibody (610943; BD Biosciences) and normalized against nuclear loading control, nucleolin (ab22758; Abcam). The membrane fraction was probed with anti-TRPC3 rabbit polyclonal antibody (ab51560; Abcam) and normalized against membrane loading control, N-cadherin (610920; BD Biosciences). ImageJ (NIH, http://rsb.info.nih.gov/ij) was used to quantify the relative band densities.

##### Dissociated primary cortical cultures

Prenatal pups (embryonic day 17-18) produced from a *Src^thl/+^* intercross were used to prepare cortical cultures according to previously published protocols (Broeke et al, 2010) with modifications. All experiments were carried out with *Src^thl/thl^* and WT littermates. Specifically, dissected cortex was digested with 0.025% Trypsin/EDTA at 37 °C in HBSS (Sigma-Aldrich) for 20 min and washed with prewarmed medium to stop digestion. Cells were triturated approximately 10 times with a 1000 μl tip. Neurons were centrifuged at 1000 rpm for 5 min, supernatant was removed and cells resuspended in culture media. Cell density was determined using an Improved Neubauer hemocytometer and low-density cultures were plated on Poly-D-Lysine (0.1 mg/ml; Sigma-Aldrich) coated glass coverslips (18 mm; #1.5, Warner Instruments) at 100,000 neurons/cm^2^. Neurons were maintained in Neurobasal medium (Invitrogen) with 2% B27 (Invitrogen), 1× Penn/Strep (Invitrogen), and 2 mM GlutaMAX (Invitrogen) at 37 °C with 5% CO_2_ for 6 days *in vitro* (DIV6).

#### Immunocytochemistry

Neurons at DIV6 were fixed with 4% paraformaldehyde/4% sucrose in PBS for 20 min, washed 3× with PBS, and permeabilized with 0.1% Triton X-100 in PBS for 20 min at room temperature (RT). Cells were incubated with specific primary antibodies overnight at 4 °C: anti-TRPC3 (rabbit polyclonal, 1:500; #NB110-74935, Novus Biologicals), anti-TFII-I (goat polyclonal, 1:50; #sc-9943 X, Santa Cruz Biotechnology), anti-NeuN (mouse monoclonal, 1:200; #MAB377, Millipore). Cells were then incubated with their respective secondary antibody: Alexa Fluor 488 goat anti-rabbit, Alexa Fluor 568 rabbit anti-goat, and Alexa Fluor 633 goat anti-mouse, (1:500; Molecular Probes) at RT for 1 h and washed 3× with PBS. Coverslips were then mounted on slides with ProLong Gold (Invitrogen) and dried in the dark at RT. When cells were incubated with secondary antibodies only, no signal was observed.

#### Image acquisition

Images were captured with an Olympus Confocal Laser Scanning Microscope FV10i-DOC with a 60× phase contrast oil-immersion objective (NA 1.35) and 488, 543, and 633 lasers. Each Z-plane was 0.3 μm. All cells were imaged at a resolution of 1024 × 1024 pixels using the same magnification and laser settings. Fluorescence intensity was measured in a region of interest over the cell and corrected for background measured outside of the cell. To test whether the fluorescence intensity of TRPC3 and TFII-I was significantly different between *Src^thl/thl^* and their WT littermates, the mean amplitude fluorescence intensity (in arbitrary units) was measured from the middle section of the entire z-stack. NeuN was used as a neuronal marker. In the figures, each bar represents the average protein level fluorescence intensity from all cells measured under that condition. Fluorescence intensity was measured using ImageJ (NIH, http://rsb.info.nih.gov/ij).

### Statistical analysis

One-way ANOVA (balance bean, grip strength, rotarod, acoustic startle, fear conditioning, shock threshold, visual object recognition), two-way ANOVA (social approach, open field, Morris water maze) or repeated-measures ANOVA (tube test, olfactory test, USVs) were used to test for the effect of genotype. If this was significant, the data was further analyzed using Tukey’s *post hoc* test. Western blot band densities were analyzed using Students *t* test and fluorescence intensity using one-way ANOVA. All figure data represents mean ± SEM. An analysis with a value of *p* < 0.05 was considered to be statistically significant.

#### Study approval

All animal procedures were approved by the Animal Management Committee of Mount Sinai Hospital, the Toronto Center for Phenogenomics, and the University of Toronto Animal Care Committee, and performed in accordance with the guidelines provided by the Canadian Council on Animal Care.

## Results

### Mutation in the *Src* gene leads to craniofacial abnormalities

During a routine observation of our animal colony, we noticed two 12-d-old “toothless” pups (one male and one female) that could be distinguished from their littermates by small size and a lack of incisors. Both parents lacked the observed phenotype, suggesting it was inherited as an autosomal recessive trait. These mice were on the mixed S129 genetic background derived from an R1 embryonic stem cell line. To identify the mutation responsible for this phenotype, we crossed the parents with C57BL6/J mice, intercrossed the resulting progeny, and performed genome-wide linkage analysis on 20 (S129xC57BL6/J) F2 “toothless” (*thl*) mice. Only one marker, D2Mit411, of S129 origin showed significant linkage with the lack of incisors trait. To identify boundaries of the critical interval region containing the mutation of interest, 10 *thl* mice were genotyped for additional markers surrounding D2Mit411 (Fig. 1*A*). This allowed us to narrow down the position of the causative mutation to an approximately 8.5 Mb interval between D2Mit285 (Ensembl position: 152857301) and rs27332130 (Ensembl position: 161510193). One of the genes within the critical interval region, *Src* (Ensembl position: 157418444), caught our attention due to a known role of Src protein in the bone remodelling process (Lowe et al, 1993). Moreover, *Src*-null mice lack incisors, similar to our *thl* mutants (Tiffee et al, 1999). To evaluate whether *thl* was a novel mutation in the *Src* gene, we sequenced the *Src* coding region and discovered the insertion of a C nucleotide into exon 12, resulting in a frame shift and premature stop codon (Fig. 1*B*). Immunoblotting of brain lysates of the *Src* ^*thl/thl*^ mice failed to detect the predicted ∼47 kDa truncated Src protein (Fig. 1*C*), implying that the mRNA was likely degraded by nonsense-mediated decay.

In addition to their small size and the lack of incisors, *Src* ^*thl/thl*^ mice had variable numbers of molar teeth (usually one or two were absent, sometimes from the upper jaw and sometimes from the mandible). Since visual inspection revealed that the *Src* ^*thl/thl*^ mice exhibited craniofacial anomalies, faxitron images were used for cephalographic analysis (Fig. 1*D*). All of the parameters analyzed showed a significant reduction in length for the *Src* ^*thl/thl*^ mice, with the exception of the Mx1-Mx2 distances, which tended to be reduced, and Go-Mn, which was not affected (Table 1). Notably, the cranial base in these mice was shortened both anteriorly and posteriorly. Defects in incisor and molar tooth formation and eruption were also evident, similar to that previously described in *Src* mutants (Soriano et al, 1991; Tiffee et al, 1999). The *Src* ^*thl/thl*^ mice were smaller in size with body weight reduction (Fig. 1*E*).

**Table 1:** Linear variables of the skull in WT and *Src^thl/thl^* mice

Measurement	Wildtype		*Src^thl/thl^*		*p* value
	Mean	SEM	Mean	SEM	
Ps-Rh	145.1	1.4	117.6	4.2	0.0008
Op-Ne	235.5	1.3	200.8	5.2	0.0007
Na-Rh	111.3	1.8	91.0	3.4	0.0018
Go-Mn	56.5	3.6	49.9	3.7	0.24
Ps-Na	77.8	0.9	65.5	1.3	0.00024
Ps-Ba	110.5	2.1	97.5	2.7	0.0092
Mx1-Mx2	61.0	0.6	56.0	2.3	0.077
Cw1-Cw2	117.3	0.5	110.8	1.9	0.017
Op-Inc	261.4	0.7	221.0	6.9	0.0011
Ps-Inc	113.1	1.4	94.4	5.1	0.013

Nine-week old male mice (n =2 WT, n= 2 *Src^thl/thl^*) were dissected to obtain the skull for faxitron analysis. Images were printed at the same magnification and the distances between established landmarks measured. Data are presented as mean ± SEM.

Lateral cephalometric landmarks: Ba: basion, the most posterior point of occipital bone anterior to the foramen magnum; Cw1 and Cw2: most lateral points of the calvarium; Go: gonion, the most posterior point of the mandibular angular process; Mn: point in the deepest part of the antegonial notch curvature; Mx1 and Mx2: most anterior and posterior points of the maxilla; Na: nasion, the junction of the frontonasal and internasal sutures; Ne: nasale, intersection of nasal bones, rostral point; Ps: pre-sphenoid, the middle of the presphenoethmoidal synchrondrosis; Inc: incisor, the most prominent point between incisal edges of lower incisors.

Dorsoventral cephalometric landmarks: Rh: rhinion, the most anterior point of the nasal bones in the midline; Op: opisthion, midsaggital point on the posterior margin of the foramen magnum.

### Social interaction and social recognition is altered in *Src* mutant mice

#### Social approach and short-term social recognition

**Figure 2.**
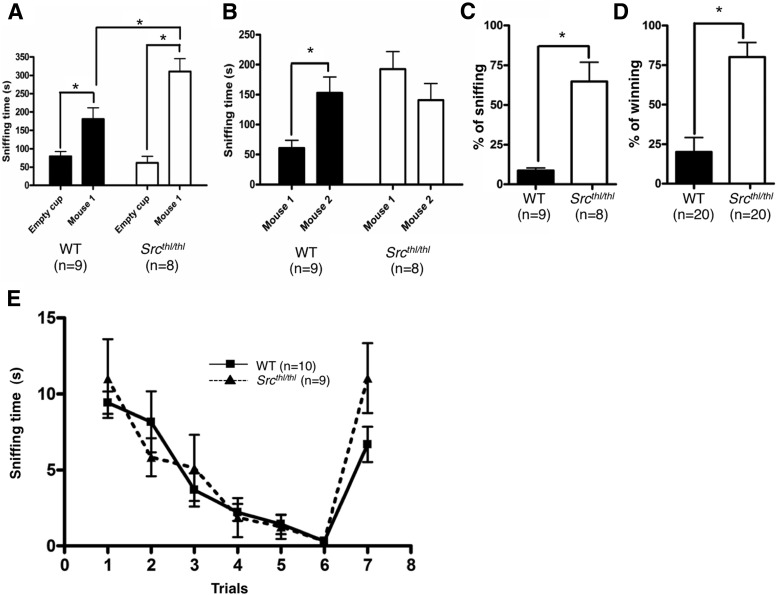
Social approach behaviors are increased and social recognition is impaired in *Src^thl/thl^* mice. ***A***, *Src^thl/thl^* mice spent more time sniffing a social cage versus a nonsocial cage (*n* = 9 WT males, *n* = 8 *Src^thl/thl^* males). ***B***, During the second phase of the test to measure social recognition, *Src^thl/thl^* mice did not show a preference for a novel mouse versus a familiar mouse from the first phase (*n* = 9 WT males, *n* = 8 *Src^thl/thl^* males). *p < 0.05, compared to the chamber with empty cage, between genotypes and within genotypes, respectively. ***C***, In a direct social interaction test, *Src^thl/thl^* mice showed an increased frequency of interactions with a stranger mouse. Data are presented as mean ± SEM (*n* = 9 WT males, *n* = 8 *Src^thl/thl^* males). *p < 0.05, between genotypes. ***D***, *Src^thl/thl^* mice showed social dominance over their WT littermates in a tube test (*n* = 20 WT males, *n* = 20 *Src^thl/thl^* males). *p < 0.05, between genotypes. ***E***, *Src^thl/thl^* mice were able to habituate to a smell over time and dishabituate toward a novel smell, demonstrating intact olfaction (*n* = 10 WT (5 males, 5 females), *n* = 9 *Src^thl/thl^* (5 males, 4 females)). Data are presented as mean ± SEM.

We used a three-chambered apparatus in which mice were tested in two different trials. In the first trial, the subject mouse was given the choice to spend time with a social object (mouse) or spend time with a nonsocial object (empty). There was a statistically significant interaction between social versus nonsocial object and genotype (*p* = 0.0089, *F*_(1,15)_ = 9.02). The effect of genotype was not quite significant (*p* = 0.052, *F*_(1,15)_ = 4.42). The effect of social versus nonsocial object was highly significant (*p* = 0.0001, *F*_(1,15)_ = 50.85). Both *Src^thl/thl^* and WT mice showed a preference for the mouse (time with mouse vs nonsocial object: WT: *p* = 0.0082, *F*_(1,16)_ = 9.11; *Src* ^*thl/thl*^ mice: *p* = 0.0001, *F*_(1,14)_ = 40.42), but the *Src^thl/thl^* mice spent significantly more time with the mouse compared to WT mice (WT = 180.6 s ± 30.89, *Src^thl^* ^/^*^thl^* = 310.6 s ± 34.91, *p* = 0.0135, *F*_(1,14)_ = 7.8) (Fig. 2*A*). However, in the test for social novelty preference, only WT mice showed a significant preference for a nonfamiliar mouse (Mouse 2) in comparison with time spent with a familiar mouse (Mouse 1) (time for Mouse 1: *Src^thl/thl^* = 60.90 s ± 13.10; time for Mouse 2: *Src^thl/thl^* = 153.1 s ± 26.30, *p* = 0.0063, *F*_(1,16)_ = 9.85) (Fig. 2*B*). *Src^thl/thl^* mice were not able to distinguish between Mouse 1 and Mouse 2, suggesting impairment in short term social recognition in *Src^thl/thl^* mice (time for familiar mouse: *Src^thl/thl^* = 192.8 s ± 29.00, time for nonfamiliar mouse: *Src^thl/thl^* = 141.0 s ± 27.56, *p* = 0.2165, *F*_(1,14)_ = 1.68) (Fig. 2*B*). Number of entries into each compartment was not different between genotypes in each session.

#### Direct social interaction test

During a 5 min direct social interaction test, *Src^thl/thl^* mice spent more time involved in active social approaches than WT mice (WT 8.73 s ± 1.66; *Src^thl/thl^*: 64.72 s ± 12.21, *p* = 0.0003, *F*_(1,16)_ = 20.66) (Fig. 2*C*).

#### Tube test of social dominance

When WT mice and *Src^thl/thl^* mice were facing each other, *Src^thl/thl^* mice showed social dominance over approximately 70% of their matches (WT = 20.00 ± 9.18; *Src^thl/thl^* = 80.00 ± 9.18, *p* = 0.0001, *F*_(1,38)_ = 21.38) (Fig. 2*D*).

#### Olfactory test

Both genotypes showed dishabituation towards a new smell on trial seven of an olfactory test, indicating an intact olfactory system (Fig. 2*E*). The effect of the genotype was not significant (*p* = 0.3915, *F*_(1,102)_ = 0.77), but the effect of trial was considered highly significant (*p* = 0.0001, *F*_(1,102)_ = 15.46).

#### Mating induced USVs

**Figure 3.**
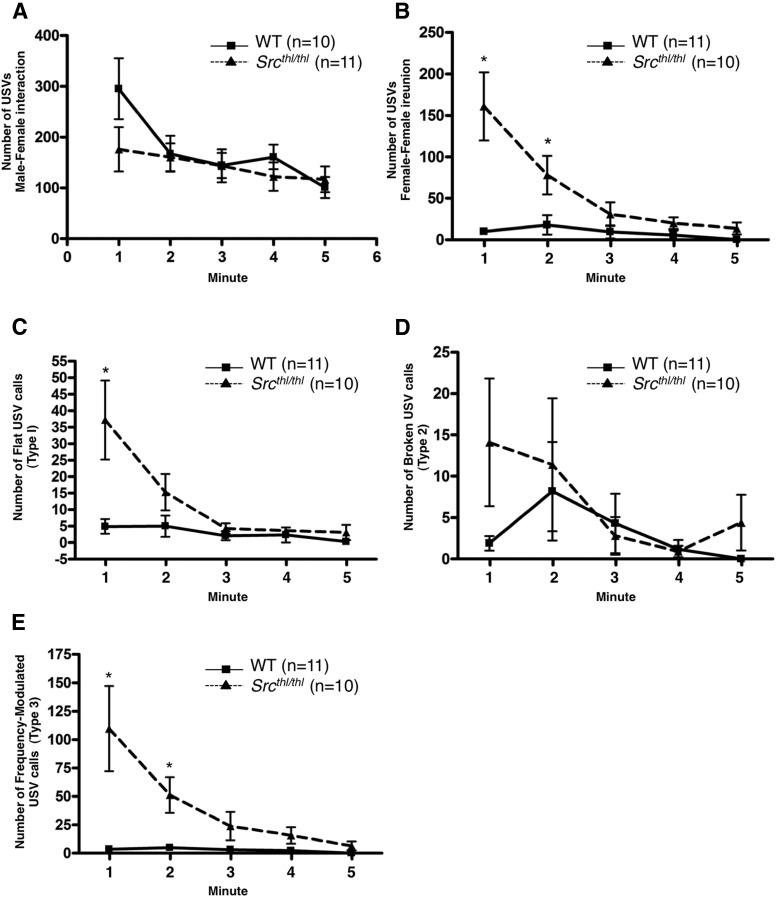
Enhanced social reunion of female *Src^thl/thl^* mutant mice. ***A***, Time course of total number of USVs in WT and *Src^thl/thl^* male mice when introduced to females for a 5 min trial. A gradual decline in total number of calls was observed across time but the total number of calls was still high, with no significant difference between WT and *Src^thl/thl^* mice. Data are presented as mean ± SEM for each time point (*n* = 10 WT, *n* = 11 *Src^thl/thl^*). ***B***, Time course of total number of USVs in WT and *Src^thl/thl^* females when reunited with a litter mate for a 5 min trial. A gradual decline in total number of calls is observed across time with a significant difference between WT and *Src^thl/thl^* females in total number of USVs produced in the first and second minutes (*n* = 11 WT, *n* = 10 *Src^thl/thl^*). ***C***, There is a significant difference in the number of flat (Type 1) USVs produced by WT versus *Src^thl/thl^* females at minute 1 (*n* = 11 WT, *n* = 10 *Src^thl/thl^*). ***D***, There was no significant difference in the number of broken (Type 2) USVs produced by WT versus *Src^thl/thl^* females across time (*n* = 11 WT, *n* = 10 *Src^thl/thl^*). ***E***, There is a significant difference in the number of frequency modulated (Type 3) USVs produced by WT versus *Src^thl/thl^* females at minutes 1 and 2 (*n* = 11 WT, *n* = 10 *Src^thl/thl^*). Data are presented as mean ± SEM for each time point. *p < 0.05 between genotypes.

*Src^thl^* ^/^*^thl^* and WT male mice emitted similar numbers of ultrasonic calls during the 5 min encounter with a female mouse. Repeated-measure ANOVA revealed no significant difference between groups across time (*p* = 0.4512) (Fig. 3*A*).

#### Social reunion induced USVs

Most of the female calls during female−female social reunion paradigm occurred in the first 2 min of the 5 min trial. A repeated-measure ANOVA over the 5 min revealed a significant difference in number of USV calls between groups (*p* = 0.0044, *F*_(1,72)_ = 10.6). The interaction between time and genotype was highly significant (*p* = 0.0001, *F*_(4,72)_ = 9.08). *Src^thl/thl^* females called more in both the first (WT = 9.27 ± 3.55; *Src^thl/thl^* = 160.9 ± 41.04, *p* = 0.001, F_(1,19)_ = 14.96) and second minute (WT = 16.64 ± 10.79; *Src^thl/thl^* = 78.00 ± 23.33, *p* = 0.0235, *F*_(1,19)_ = 6.06) when compared to WT females (Fig. 3*B*).

A difference was observed in the number of both flat (Type 1) and frequency-modulated (Type 3) USVs between WT and *Src^thl/thl^* female mice (types of calls were described previously by Wang et al, 2008). A repeated-measures ANOVA over the 5 min of the number of Type 1 USV calls revealed a significant difference in the number of calls between groups (*p* = 0.0219, *F*_(1,72)_ = 6.29) and the interaction between time and genotype was highly significant (*p* = 0.0009, *F*_(4,72)_ = 5.29). Type 1 calls were significantly higher in *Src^thl/thl^* mice during the first minute of recording only (WT = 4.55 ± 2.06; *Src^thl/thl^* = 37.20 ± 11.99, *p* = 0.0111, *F*_(1,19)_ = 7.91) (Fig. 3*C*). No significant effect of genotype (*p* = 0.4164, *F*_(1,72)_ = 0.69) or time (*p* = 0.3252, *F*_(4,72)_ = 1.18) was observed between groups for Type 2 calls (Fig. 3*D*). However, Type 3 USV calls showed a significant genotype effect between groups (*p* = 0.0084, *F*_(1,72)_ = 8.75) and the interaction between time and genotype was highly significant (*p* = 0.0002, *F*_(4,72)_ = 6.29). The number of calls were significantly higher in *Src^thl/thl^* female mice during the first (WT = 3.27 ± 1.14; *Src^thl/thl^* = 109.6 ± 37.53, *p* = 0.0077, *F*_(1,19)_ = 8.86) and second minute (WT = 4.64 ± 2.44; *Src^thl/thl^* = 51.30 ± 15.76, *p* = 0.0063, *F*_(1,19)_ = 9.42) (Fig. 3*E*).

## Exploratory activity and anxiety

**Figure 4.**
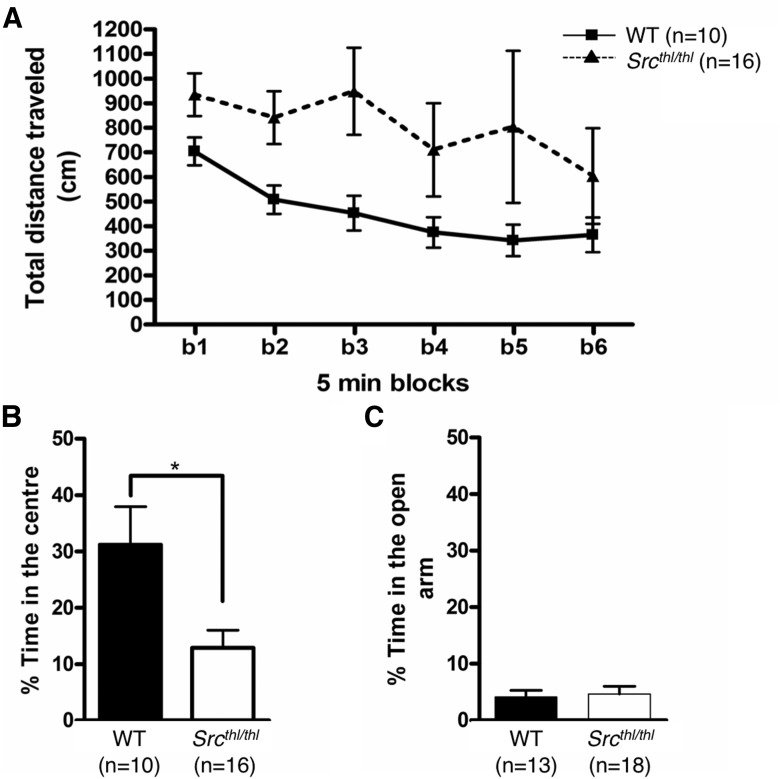
*Src^thl/thl^* mutant mice show hyperactivity in the open field. ***A***, *Src^thl/thl^* mutant mice showed an increase in total distance travelled in the open field compared with WT mice (*n* = 10 WT (5 males, 5 females), *n* = 16 *Src^thl/thl^* (9 males, 7 females)). ***B***, Percentage of time spent in the center of the arena versus the entire open field was decreased in *Src^thl/thl^* (*n* = 10 WT (5 males, 5 females), *n* = 16 *Src^thl/thl^* (9 males, 7 females)). *p < 0.05 between genotypes. ***C***, Percentage time spent in the open arms versus the closed arms of the elevated plus maze showed no significant differences between genotypes (*n* = 13 WT (7 males, 6 females), *n* = 18 *Src^thl/thl^* (9 males, 9 females)). Data are presented as mean ± SEM.

In the open-field test, there was a significant effect of genotype (*p* = 0.0381, *F*_(1,170)_ = 4.66) and time interval (*p* = 0.0006, *F*_(5,170)_ = 4.54) on the total distance travelled (Fig. 4*A*), with the *Src^thl/thl^* mice having a greater total distance travelled and both genotypes travelling more during the first 15 min. *Src^thl/thl^* mice travelled less distance in the center of the arena compared with WT mice (WT = 31.19 ± 6.76%; *Src^thl/thl^* = 12.89 ± 3.16%, *p* = 0.011, *F*_(1,24)_ = 7.59, one-way ANOVA) (Fig. 4*B*), suggesting an increase in anxiety-like behavior in the *Src^thl/thl^* mice. However, we did not observe any difference between genotypes in the elevated plus maze, another test for anxiety. The percentage time spent in the open arm was similar in both groups (WT = 4.11 ± 1.17%; *Src^thl/thl^* = 4.64 ± 1.35%, *p* = 0.7775, *F*_(1,29)_ = 0.08, one-way ANOVA) (Fig. 4*C*). The total distance travelled in the elevated plus maze was not different between the two genotypes.

## Sensory and motor function

**Figure 5.**
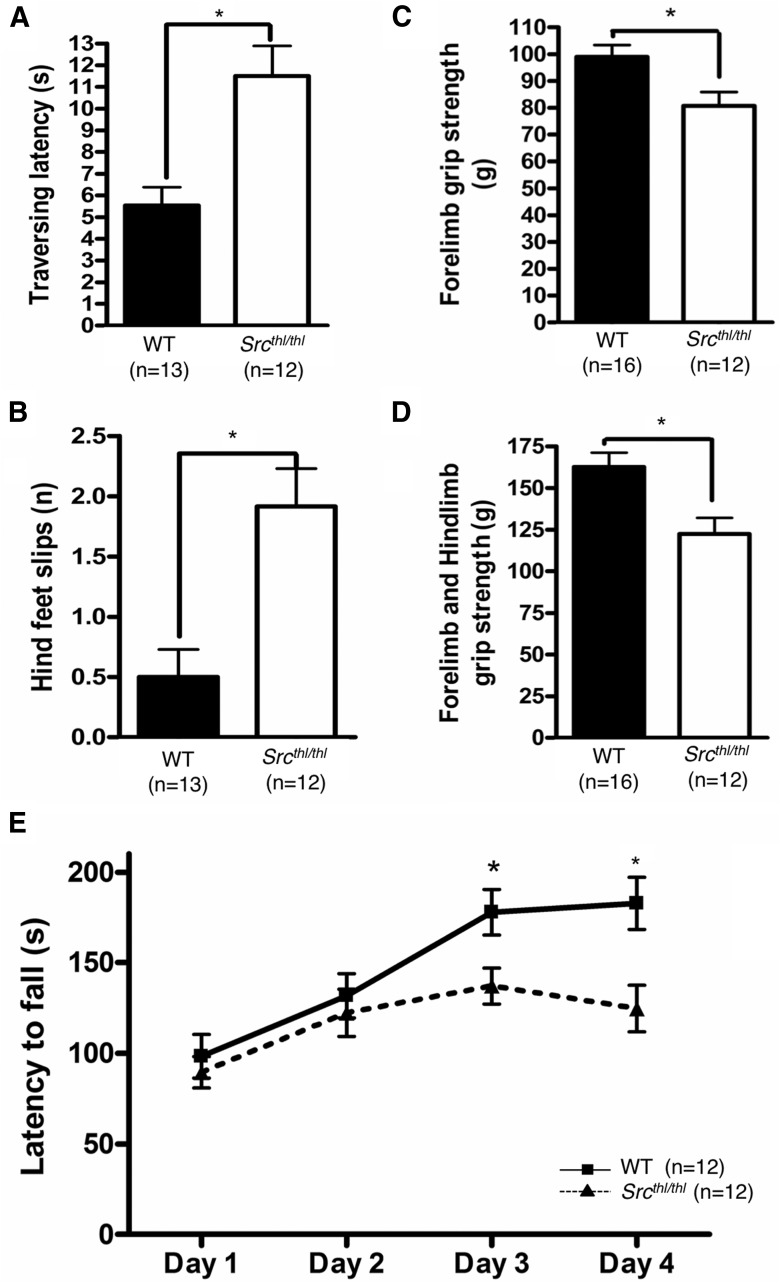
Balance and motor strength deficiency in *Src^thl/thl^* mice and the inability to learn motor skills. Quantitative analysis of raised balance beam, grip strength, and rotarod task in WT and *Src^thl/thl^* mice. ***A***, *Src^thl/thl^* mice needed significantly more time to cross the balance beam than did WT mice (*n* = 13 WT (7 males, 6 females), *n* = 12 *Src^thl/thl^* (6 males, 6 females)). ***B***, *Src^thl/thl^* mice had significantly more hindfoot slips than did WT mice. ***C***, Grip strength analysis revealed significantly reduced forelimb grip strength in *Src^thl/thl^* mice compared with WT animals (*n* = 16 WT (9 males, 7 females), *n* = 12 *Src^thl/thl^* (6 males, 6 females)). ***D***, Grip strength analysis revealed significantly reduced combined forelimb and hindlimb grip strength in *Src^thl/thl^* mice compared with WT animals (*n* = 16 WT (9 males, 7 females), *n* = 12 *Src^thl/thl^* (6 males, 6 females)). ***E***, Mice were tested on an accelerating rotarod with the same speed for 4 consecutive days. The latencies from rotation onset until the mice fell off the rod were measured. WT mice managed to stay significantly longer on the accelerating rotarod than *Src^thl/thl^* mice on days 3 and 4. The falling latencies were similar in both groups on days 1 and 2. Falling latencies were compared within genotype between days 1 and 4 (*n* = 12 WT (6 males, 6 females), *n* = 12 *Src^thl/thl^* (6 males, 6 females)). Data are presented as mean ± SEM. *p < 0.05.

We assessed motor coordination, strength, and motor skill learning in *Src^thl/thl^* mice using the balance beam, grip strength, and rotarod tasks. *Src^thl/thl^* mice took significantly longer time to cross the beam (WT = 5.54 ± 0.84 s; *Src^thl/thl^* =11.50 ± 1.39 s, *p* = 0.0011, *F*_(1,23)_ = 13.93) and had increased number of foot slips while crossing the beam (WT = 0.50 ± 0.23; *Src^thl/thl^* =1.92 ± 0.31, *p* = 0.0014, *F*_(1,23)_ = 13.30) (Fig. 5*A*,*B*) (one-way ANOVA).

We also tested grip strength in the same mice to determine if differences in muscle strength might account for the differences in the balance beam results. This test revealed a significant decrease of grip strength for both forelimb (WT = 98.97 ± 4.37 g; *Src^thl/thl^* = 80.69 ± 5.20 g, *p* = 0.0019, *F*_(1,26)_ = 7.32) and for forelimb and hindlimb combined (WT = 162.7 ± 8.62 g; *Src^thl/thl^* = 122.5 ± 9.60 g, *p* = 0.0047, *F*_(1,26_) = 9.56) in *Src^thl/thl^* mice (Fig. 5*C*,*D*) (one-way ANOVA).

We found an overall significant difference in the amount of time mice spent walking on the rotating rod on days 3 and 4 (Day 3: WT = 177.9 ± 12.52 s; *Src^thl/thl^* = 137.1 ± 10.02 s, *p* = 0.019, *F*_(1,22)_ = 0.019; Day 4: WT = 182.7 ± 14.50 s; *Src^thl/thl^* = 124.7 ± 12.78 s, *p* = 0.0065, *F*_(1,22)_ = 9.02) but not on days 1 or 2 (Day 1: WT = 98.42 ± 11.97 s; *Src^thl/thl^* = 89.58 ± 8.70 s, *p* = 0.5566, *F*_(1,22)_ = 0.09; Day 2: WT = 131.6 ± 12.26 s; *Src^thl/thl^* = 126.2 ± 12.66 s, *p* = 0.7615, *F*_(1,22)_ = 0.36) (one-way ANOVA). This suggests a role for Src in rotarod learning rather than rotarod performance (Fig. 5*E*).

## Acoustic startle response

**Figure 6.**
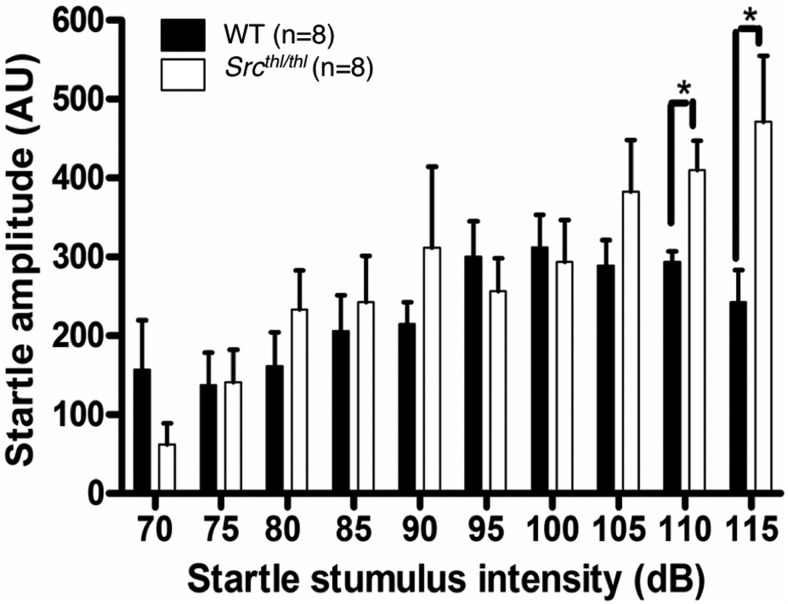
Acoustic startle in response to high startling stimulus is increased in *Src^thl/thl^* mice. Both the WT and *Src^thl/thl^* mice exhibited similar startle amplitude responses to the low-intensity acoustic stimuli (70-105 dB). *Src^thl/thl^* mice showed higher responses to the high-intensity stimuli (110-115 dB) (*n* = 8 WT (8 males), *n* = 8 *Src^thl/thl^* (18 males)). *p < 0.05.

*Src^thl/thl^* mice showed a significant enhancement of startle response at the 115 dB (WT = 242.0 ± 41.36 AU; *Src^thl/thl^* = 471.1 ± 83.82 AU, *p* = 0.028, *F*_(1,14)_ = 6.01) and 110 dB (WT = 293.2 ± 13.69 AU; *Src^thl/thl^* = 410.3 ± 36.92 AU, *p* = 0.01, *F*_(1,14)_ = 8.85) levels of startling stimulus. However, at the 70-105 dB levels, *Src^thl/thl^* mice exhibited a similar startle magnitude when compared with WT mice (*p* = 0.05) (one-way ANOVA) (Fig. 6).

## Learning, memory, and attention deficits

### Morris water maze

**Figure 7.**
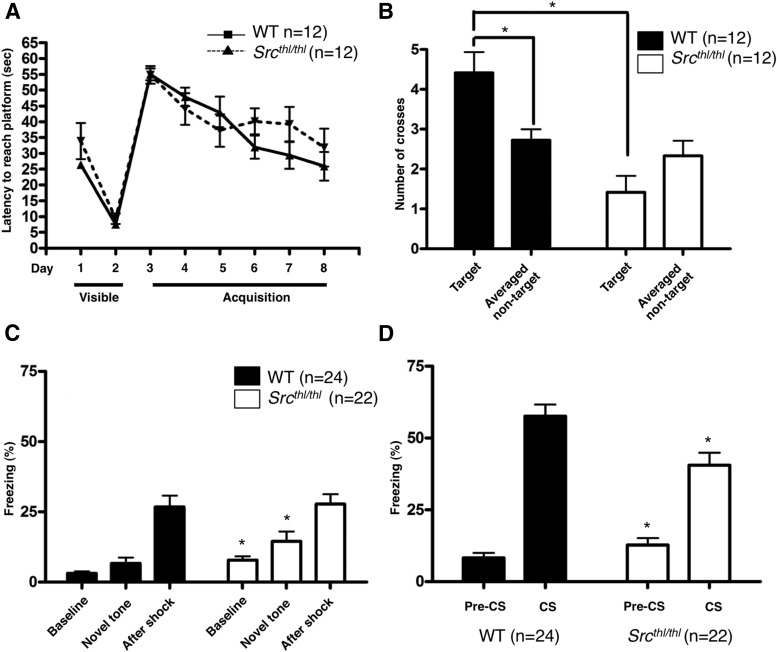
Impaired memory in *Src^thl/thl^* mutant mice. ***A***, Mice were assessed in the Morris water maze procedure (*n* = 12 WT (12 males), *n* = 12 *Src^thl/thl^* (12 males)). Mean latency to reach a target platform in a visible platform session (days 1 and 2), and in a hidden-platform acquisition phase (days 3-8). ***B***, Spatial memory retention was assessed in the probe trials administered 2 h after day 8 (*n* = 12 WT (12 males), *n* = 12 *Src^thl/thl^* (12 males)). Mean number of platform crosses during probe trial 1 are shown. *p < 0.05. ***C***, Freezing (as a percentage of total time) in a novel conditioning chamber (*n* = 24 WT (13 males, 11 females), *n* = 22 *Src^thl/thl^* (12 males, 10 females)). After recording baseline freezing for 120 s, a tone was presented for 30 s (novel tone), after which mice were given a footshock and left inside the chamber for another 30 s (after shock). A subtle increase in freezing was observed during baseline and novel tone presentation in *Src^thl/thl^* mice. *p < 0.05. ***D***, Cued fear memory 24 h after pairing (*n* = 24 WT (13 males, 11 females), *n* = 22 *Src^thl/thl^* (12 males, 10 females)). Pre-CS is freezing in the absence of the tone in a novel context, while CS is percentage freezing during tone presentation. *Src^thl/thl^* mice showed impaired auditory fear conditioning, however, context was not affected. Data are presented as mean ± SEM, *p < 0.05.

Visuospatial learning and memory was assessed using the Morris water maze task. *Src^thl/thl^* mice did not show impaired motor performance or decreased motivation to escape, as all mutants showed similar escape latencies in the visible and the hidden platform task (*p* = 0.4648, two-way ANOVA) (Fig. 7*A*).There was a significant effect of genotype (*p* = 0.001, *F*_(1,22)_ = 22.32) but no effect of target (*p* = 0.3926, *F*_(1,22)_ = 0.76) (two-way ANOVA) in the probe trial given after 6 d of training. WT mice showed significantly more crossings of the target platform position compared with similarly sized and positioned areas in the other quadrants (crossing in target platform quadrant: 4.42 ± 0.514, averaged crossing in nontarget quadrants: 2.72 ± 0.27, *p* = 0.0082, *F*_(1,22)_ = 8.44), whereas *Src^thl/thl^* mice did not show any preference for the target location (crossing in target platform quadrant: 1.42 ± 0.42, averaged crossing in nontarget quadrants: 2.33 ± 0.38, *p* = 0.1167, *F*_(1,22)_ = 2.67) (Fig. 7*B*).

### Fear conditioning

Levels of freezing in response to both a context- and cued-fear CS was measured. During the context test, *Src^thl/thl^* mice displayed similar periods of freezing relative to WT mice. In the CS test, *Src^thl/thl^* mice displayed shorter freezing time relative to WT. These findings suggest that *Src^thl/thl^* mice have impaired learning and/or memory performance. Cued fear conditioning is the most widely used behavioral task to assess amygdala function (Phelps and LeDoux, 2005). *Src^thl/thl^* mice showed impaired fear-related learning during cued fear conditioning. Mice were conditioned to two pairs of a tone (CS) and a footshock (US) on the training day. Prior to pairing, there was a moderate difference between groups in baseline freezing (WT *=* 3.14 ± 0.73%; *Src^thl/thl^* = 7.87 ± 1.32%, *p* = 0.0026, *F*_(1,44)_ = 10.21, one-way ANOVA) and freezing in response to unpaired tone (WT = 5.79 ± 1.69%; *Src^thl/thl^* = 13.97 ± 2.74%, *p* < 0.05, *F*_(1,44)_ = 6.70, one-way ANOVA). However, the post-shock freezing was similar between groups (WT = 29.21 ± 3.93%; *Src^thl/thl^* = 28.98 ± 3.24%, *p* = 0.9633, *F*_(1,44)_ = 0.04) (Fig. 4*C*), suggesting that the *Src* mutation alters general anxiety to some extent. At 24 h after pairing, the mice were tested for long-term contextual and cued fear memory. Subjects were placed in a novel chamber for 3 min prior to the presentation of the tone; both groups of mice displayed only weak freezing in the novel chamber (WT = 5.79 ± 1.69%; *Src^thl/thl^* = 13.97 ± 2.74%, *p* ≤ 0.0130, *F*_(1,44)_ = 6.70, one-way ANOVA) (Fig. 7*C*). During the tone delivery, *Src^thl/thl^* mice demonstrated weaker freezing (38.48 ± 4.26%) in comparison to WT mice (57.68 ± 3.98%, *p* = 0.0019, *F*_(1,47)_ = 10.88) (Fig. 7*D*), suggesting that Src function is important for long-term memory of the CS–US association. In contrast, contextual fear was not effected in *Src^thl/thl^* mice (WT = 50.14 ± 5.65%; *Src^thl/thl^* = 42.08 ± 4.50%, *p* = 0.2728, *F*_(1,47)_ = 1.23, one-way ANOVA).

### Shock threshold

Because pain sensitivity can also affect the strength of associative learning, we measured pain threshold [data not shown, *n* = 10 WT (5 males, 5 females), *n* = 10 *Src^thl/thl^* (5 males, 5 females)]. The minimum footshock intensity required to produce an audible vocalization response was unaltered in *Src^thl/thl^* mice. (WT = 0.29 ± 0.01 s; *Src^thl/thl^* = 0.29 ± 0.01 s, *p* = 0.5560, *F*_(1,18)_ = 0.36, one-way ANOVA), suggesting that they have similar sensitivity to painful stimuli.

### Visual object and novelty recognition

**Figure 8.**
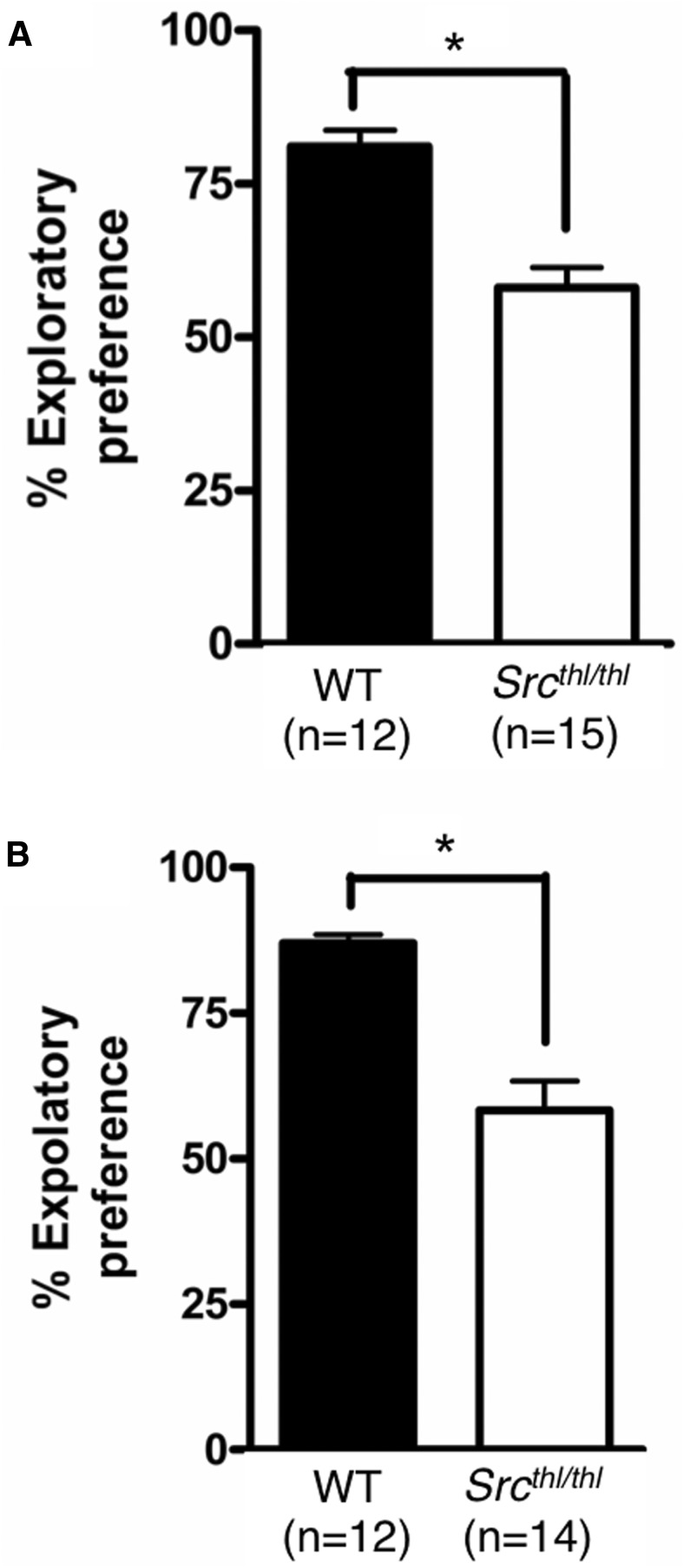
*Src^thl/thl^* mice have a deficit in short term object recognition. ***A***, Mean time (s) spent exploring the displaced versus nondisplaced objects in the spatial change session (*n* = 12 WT (6 males, 6 females), *n* = 15 *Src^thl/thl^* (8 males, 7 females)). ***B***, Mean time spent exploring the novel item versus three familiar objects in the nonspatial change session (*n* = 12 WT (6 males, 6 females), *n* = 14 *Src^thl/thl^* (7 males, 7 females). Data are expressed as mean ± SEM, *p < 0.05.

The exploration time of displaced objects and novel objects was evaluated. *Src^thl/thl^* mice demonstrated an inability to selectively react to a spatial and novelty change in the environment. There was a main effect of genotype, with an increase in exploratory preference for spending more time with the displaced object (>50%) than the nondisplaced objects displayed by WT mice, whereas *Src^thl/thl^* mice explored both object categories for a similar amount of time (∼50%) (WT = 81.08 ± 2.61%; *Src^thl/thl^* = 58.11 ± 3.20%, *p* = 0.0001, *F*_(1,25)_ = 28.81, one-way ANOVA) (Fig. 8*A*). In the novel object session, when one of the objects was substituted, *Src^thl/thl^* mice demonstrated an inability to selectively react to a novelty change. There was a main effect of genotype (WT = 87.06 ± 1.45%; *Src^thl/thl^* = 58.31 ± 5.02%, *p* = 0.0001, *F*_(1,24)_ = 26.39, one-way ANOVA) (Fig. 8*B*).

## Cellular localization of TFII-I and TRPC3

### Western blot analysis

**Figure 9.**
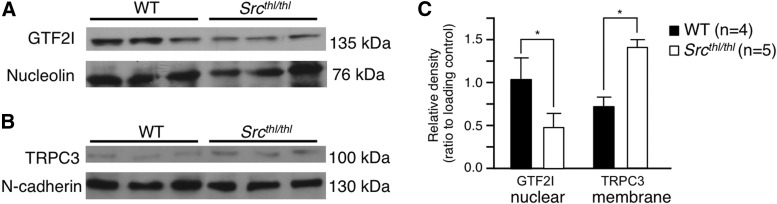
Altered cellular localization of TFII-I and TRPC3 in *Src^thl^* ^/^*^thl^* mouse brain tissue. Western blot analysis was carried out on fractionated cell lysates from WT and *Src^thl/thl^* adult mouse whole brain. Representative blots are shown (*n* = 4 WT, *n* = 5 *Src^thl/thl^*). ***A***, The nuclear fraction was probed with TFII-I antibody, with nucleolin as a loading control. ***B***, The membrane fraction was probed with TRPC3 antibody, with n-cadherin as a control. ***C***, Quantification of the Western blots. Data are expressed as mean ± SEM, *p < 0.05.

We performed Western blot analysis of fractionated cellular compartments to compare the distribution of TFII-I and TRPC3 within the cell. Since TFII-I has been shown to require phosphorylation by Src to translocate to the nucleus in transformed cell lines (Cheriyath et al, 2002), we quantified TFII-I within the nuclear fraction of whole brain from WT and *Src^thl/thl^* mice. There was significantly less TFII-I localized to the nucleus in cells from the *Src^thl/thl^* mice compared with WT mice (quantification relative to loading control, WT = 1.03 ± 0.26; *Src^thl/thl^* = 0.44 ± 0.11, Student’s *t* test *p* < 0.05; Fig. 9*A*,*C*). TRPC3 is cycled to the plasma membrane through a process that is regulated by phosphorylated TFII-I within the cytoplasm (Caraveo et al, 2006). We found significantly more TRPC3 contained within the membrane fraction in cells from the *Src^thl/thl^* mice compared with WT mice (quantification relative to loading control, WT = 0.69 ± 0.14; *Src^thl/thl^* = 1.44 ± 0.16, Student’s *t* test *p* < 0.05; Fig. 9*B*,*C*).

#### Immunocytochemistry

**Figure 10.**
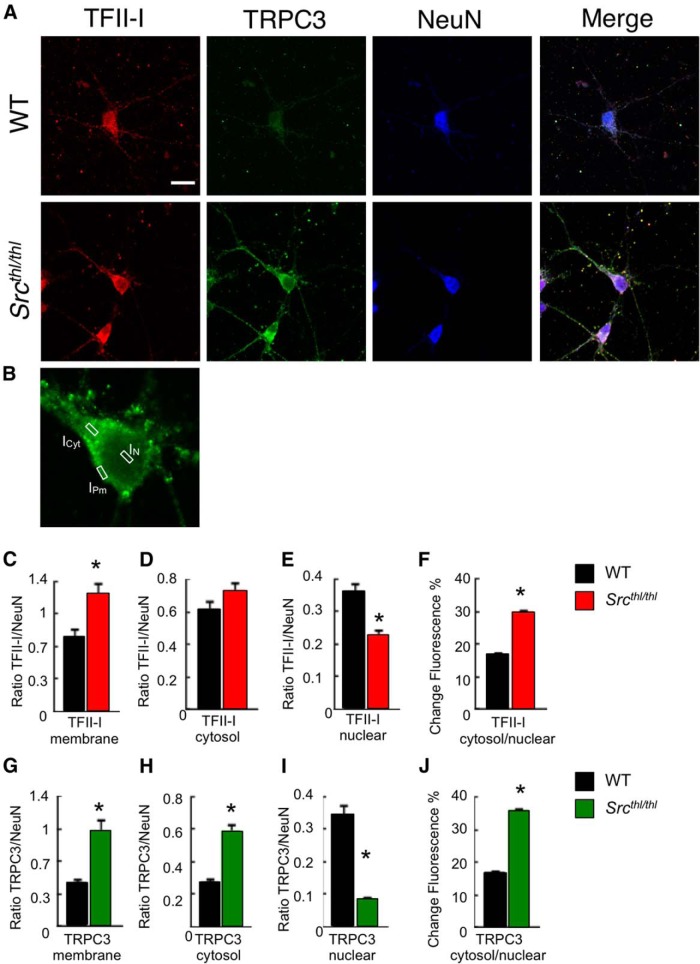
Altered cellular localization of TRPC3 and TFII-I in cultured cortical neurons. TFII-I and TRPC3 fluorescence intensity was calculated from pooled data derived from two independent experiments, each using 10 pups from two different litters (*n* = 60). ***A***, DIV6 cortical neurons were labeled for TFII-I in red, TRPC3 in green, and NeuN in blue. Scale bar is shown in the first panel, WT TFII-I, 40 μm. ***B***, TFII-I, TRPC3, and NeuN fluorescence intensity was measured in the nucleus (I_N_), cytosol (I_Cyt_) and plasma membrane (I_Pm_). Fluorescence intensity was measured in a single section in the middle of the entire Z-stack for every neuron imaged. Scale bar, 5 μm. ***C***, *Src^thl/thl^* mice had higher levels of TFII-I in the membrane than WT littermates. ***D***, There was no significant differential expression of TFII-I in the cytosol. ***E***, WT mice showed higher levels of TFII-I in the nucleus compared to *Src^thl/thl^* littermates. ***F***, The 30% difference in TFII-I levels between the cytoplasm and nucleus in *Src^thl/thl^* mice and 17% difference in WT mice shows that TFII-I was more evenly distributed throughout the cell in WT mice. ***G***, *Src^thl/thl^* mice had higher levels of TRPC3 in the membrane than WT littermates. ***H***, *Src^thl/thl^* mice had less TRPC3 in the cytosol than WT littermates. ***I***, *Src^thl/thl^* mice had less TRPC3 in the nucleus compared to WT littermates. ***J***, There was a 36% difference in TRPC3 levels between the cytosol and the nucleus in *Src^thl/thl^* mice. In WT mice, there was only a 17% difference of fluorescence, which means that TRPC3 was more evenly distributed throughout the cell in WT mice. Data are expressed as mean ± SEM, *p < 0.05.

To support our findings of altered TFII-I and TRPC3 cellular localization by Western blot analysis, we performed immunocytochemistry and confocal imaging on primary cortical neurons from *Src^thl/thl^* mice and their WT littermates, and compared the expression of TRPC3 and TFII-I in the membrane, cytosol, and nucleus (Fig. 10*A*,*B*). We found that TFII-I was significantly less abundant in the nucleus (Fig. 10*E*, *p* = 0.00007, *F* = 1.42, one-way ANOVA) in *Src^thl/thl^* mice but showed higher expression in the cell membrane (Fig. 10*C*, *p* = 0.001, *F* = 11.98, one-way ANOVA). TFII-I levels in the cytosol were not significantly different between *Src^thl/thl^* mice and their WT littermates (Fig. 10*D*, *p* = 0.237, *F* = 1.42, one-way ANOVA). WT mice showed a broad expression of TFII-I in the nucleus and cytosol, as evidenced by only a 17% difference in fluorescence between the nucleus and cytoplasm, whereas *Src^thl/thl^* mice showed a 30% difference in fluorescence (Fig. 10*F*, *p* = 0.014, *F* = 6.4, one-way ANOVA).

There was also a significant difference in TRPC3 expression between *Src^thl/thl^* and WT mice. *Src^thl/thl^* mice had an increased level of TRPC3 in both membrane and cytosol (Fig. 10*G*: *p* = 0.00001, *F* = 22; Fig. 10*H*: *p* = 0.0000007, *F* = 31, one-way ANOVA), in contrast with the low expression of TRPC3 in the nucleus (Fig. 10*I*, *p* = 0.0000000000003, *F* = 79, one-way ANOVA). WT mice showed only a 17% difference in fluorescence between the nucleus and cytoplasm, whereas *Src^thl/thl^* mice showed a 36% difference in fluorescence (Fig. 10*J*, *p* = 0.00000000001, *F* = 74, one-way ANOVA).

## Discussion

Disruption of both upstream and downstream components of the Src signaling pathway have been linked with human genetic disorders whose behavioral phenotypes include alterations in social interaction and cognition. We hypothesized that altered Src function could contribute to phenotypes that overlap with those found in WBS or autism spectrum disorder (ASD), and that these phenotypes might, at least in part, be due to altered TFII-I phosphorylation by Src. Here we report that mice lacking Src exhibit a complex behavioral and cognitive phenotype that does indeed exhibit overlap with WBS.

The *Src* mutant mice show craniofacial abnormalities and growth retardation, both apparent in WBS. The mice have a short cranial base and defects in incisor and molar tooth formation and eruption. Individuals with WBS have a shortened posterior cranial base and thickened frontal and occipital bones (Axelsson et al, 2005), reminiscent of the thickened calvaria and osteopetrosis observed in *Src* mutants (Soriano et al, 1991). Tooth development is also aberrant in WBS. Tooth shape is tapered and the teeth are usually more widely spaced, with ∼40% of individuals having agenesis of at least one permanent tooth, and 12% having at least six missing teeth (Axelsson, 2005). Mice with hemizygous deletion of *Gtf2i* do not show an overt craniofacial abnormality or tooth defects (Lucena et al, 2010; Sakurai et al, 2011), however, it is possible that altered TFII-I phosphorylation due to lack of Src has a larger impact than *Gtf2i* hemizygosity. Unfortunately, *Gtf2i* homozygous null embryos die well before tooth-bud formation with neural tube defects and exencephaly (Enkhmandakh et al, 2009; Sakurai et al, 2011), making phenotypic assessment of tooth or craniofacial development in animals with lower levels of TFII-I impossible. In mice, all GTF2I family member genes (*Gtf2i*, *Gtf2ird1*, and *Gtf2ird2*) showed strong expression in the developing teeth (Ohazama and Sharpe, 2007), and evidence from both mice and humans suggest that GTF2IRD1 is involved in craniofacial development (Tassabehji et al, 2005; Howard et al, 2012), suggesting that there could be downstream effects in common with Src.

We also observed differences in social behavior in the *Src^thl/thl^* mice that were similar to those observed in people with WBS. Sociability was increased in four different social affiliation tasks in *Src^thl/thl^* mice (direct social interaction, social approach, tube test, and social reunion), suggesting that Src plays a significant role in modulating social interaction. Interestingly, although the *Src^thl/thl^* mice showed an increased social dominance in the tube test, this was unlikely the result of increased aggression, since we often observed the *Src^thl/thl^* mice standing passively in the middle of the tube, looking at the WT mice (personal observation). The WT mice tried to escape by moving under the *Src* mutant mice, however, and after several unsuccessful attempts they backed out of the tube. This finding is in agreement with a study that found social motivation in children with WBS was not driven by a desire for social dominance, unlike in typically developing children (Ng et al, 2014). In the social reunion of female mice paradigm, there was an increase in the number of calls produced by *Src^thl/thl^* females in the first 2 min of the test, compared to WT females, whereas the number of USVs was unchanged in the mating-induced USV experiment, indicating that loss of Src is related to increased social interest but not reward-seeking behaviors such as courtship and mating (Gourbal et al, 2004; Moles et al, 2007; Lahvis et al, 2011).

The *Src* mutant mice showed increased social approach, which may be similar to the increased social approach documented in individuals with WBS (Doyle et al, 2004). However, they failed to show a preference for the novel mouse in the social short-term recognition section of this task, unlike individuals with Williams syndrome who have a heightened approachability toward strangers (Doyle et al, 2004). Lack of preference for a novel mouse is usually an indication of impaired social recognition, but in this case it may reflect a general problem with cognitive function, as evidenced by their failure to show a preference for a novel object or an object that has moved positions (Fig. 8). Mice heterozygous for *Gtf2i* also showed deficits in novel object recognition and social habituation, as well as increased social interaction (Sakurai et al, 2011), similar to the *Src^thl/thl^* mice.

The possible link between Src function and social interaction is intriguing. Src itself is tightly regulated by c-Src tyrosine kinase (CSK), which inhibits Src function through phosphorylation of C-terminal tyrosine residues (Okada et al, 1991; Nada et al, 1993). Interestingly, *CSK* lies within a region of human chromosome 15q24 that is deleted in some individuals with ASD (Marshall et al, 2008; McInnes et al, 2010). ASD has also been reported in individuals with duplication of the WBS region (7q11.23 duplication syndrome, Dup7) (Berg et al, 2007; Van der Aa et al, 2009; Levy et al, 2011; Sanders et al, 2011) and a *GTF2I* intronic haplotype has been associated with ASD (Malenfant et al, 2012). It could be hypothesized, therefore, that upregulation of TFII-I function, either through gene duplication or through disruption of Src inhibition, might contribute to decreased social interaction.

We assessed exploratory activity and anxiety-related behaviors using the open-field test and the elevated plus maze. *Src^thl/thl^* mice showed increased activity, a common finding in WBS (Mervis and John, 2010), but the anxiety assessment was inconclusive: they spent significantly less time in the center of the open field compared to WT littermates, but showed no genotype differences in the plus maze. In the startle response, *Src^thl/thl^* mice exhibited greater reaction to high-intensity stimuli (110 dB and 115 dB) than WT mice, suggesting either sensitivity to loud noises (decreased acoustic threshold) or a decrease in the sound level necessary to provoke a startle response (decreased response threshold). The elevated startle response could arise from an abnormality in secondary brain regions that modulate the primary startle response, either through intrinsic disturbance such as altered synaptic connections, or indirectly, through an increase in arousal, rather than a change in the primary sensory response of the auditory circuit. The presentation of over-responsiveness to sensory stimuli in individuals with WBS supports a potential role for Src signaling in the modulation of sensory input and the acoustic startle reflex. Studies in individuals with WBS have suggested that the hypersensitivity to sound may be due to hyperexcitability of the auditory efferent system, coupled with diminished or absent acoustic reflexes (Attias et al, 2008).

Intellectual disability is common in WBS, but some cognitive abilities are more affected than others, and in particular deficits in visuospatial constructive cognition are a hallmark of WBS (Mervis, 1999). Impairment in visuospatial learning and memory of *Src* mutant mice was apparent in the probe trial given after 6 d of training, where the mice showed a significant decrease in the number of crossings over the learned position of the platform compared to WT mice (Fig. 7*B*). The smaller size of the mice could make comparison to WT littermates problematic in behavioral paradigms where motor performance is important. The mutant mice had mild deficits in motor performance and learning when assessed on the rotarod and balance beam (Fig. 5), suggesting that they could be experiencing fatigue due to greater muscle weakness. Alternatively, since their rotarod performance was equivalent to WT mice for the first 2 d of training, the impairment may be in motor learning rather than motor performance. The mutant mice showed similar escape latencies in the visible and hidden platform task of the water maze test, and their swim speed was similar to that of WT mice (Fig. 7*A*), suggesting that the visuospatial learning deficit was not due to impaired motor performance or decreased motivation to escape. Spatial learning and memory has been linked with hippocampal function in mice (Phelps and LeDoux, 2005), but in WBS, functional imaging has linked visuospatial construction deficits with abnormal processing in the dorsal stream around the intraparietal sulcus (Meyer-Lindenberg et al, 2004).

Amygdala dysfunction has also been demonstrated in WBS using functional imaging, and *Src* mutant mice exhibited impaired cued fear conditioning, which is dependent primarily upon amygdala-based learning, though contextual fear conditioning was unaffected, suggesting relatively intact hippocampal function (Phelps and LeDoux, 2005; Kim and Jung, 2006). Src is an abundant tyrosine kinase that phosphorylates, and therefore regulates, numerous proteins (Bromann et al, 2004), including NMDA receptors, which it regulates through phosphorylation and subsequent surface expression of NMDA receptor 2 B (NR2B) (Lu et al, 1998; Zhang et al, 2008). It has been previously demonstrated that inhibition of Src through injection of a peptide that blocks its interaction with NMDA receptors also results in deficits in amygdala-dependent learning and long-term potentiation (Sinai et al, 2010), suggesting that in the *Src* mutant mice, this deficit is due to reduced surface expression of NR2B.

Src has been shown to phosphorylate TFII-I on tyrosines 248 and 611, thereby both translocating it to the nucleus and making it transcriptionally active (Cheriyath et al, 2002). Since the *Src* mutant mice showed overlap in phenotype with individuals who have WBS, we investigated TFII-I subcellular localization and found a >50% increase in TFII-I localized to the plasma membrane, as well as a decrease of ∼40% from WT levels in the nucleus of neurons from *Src^thl/thl^* mice (Figs. 9, 10). These findings are in direct contrast to previous *in vitro* data that showed increased nuclear localization of TFII-I upon inactivation of Src (Cheriyath et al, 2002). However, our experiments were carried out in primary neuronal cells rather than transformed fibroblast cell lines, and there are likely to be differences in Src function and TFII-I regulation between different cell types, and between primary cells and transformed lines. Our findings suggest that lack of phosphorylation by Src has a direct effect on the cellular localization of TFII-I, resulting in altered protein levels in both the nucleus and the cell membrane.

Whilst in the cytoplasm, TFII-I regulates membrane localization of TRPC3 through binding to and sequestering of PLC-γ, which usually shuttles TRPC3 subunits to the plasma membrane where they form a channel and allow agonist-inducible calcium entry (Caraveo et al, 2006). *Src^thl/thl^* mice showed an increase in membrane-bound TRPC3 in whole-brain lysate (*p* < 0.05; Fig. 9*B*,*C*) and in primary cultured neurons (Fig. 10*G*), which mirrors the predicted state in WBS, where less TFII-I is available in the cell, resulting in more availability of PLC- γ.

Given that we found less TFII-I in the nucleus, and apparently unaltered levels of TFII-I in the cytosol (Fig. 10*A*,*D*), the finding of more TRPC3 in the membrane is somewhat counterintuitive. However, the binding of TFII-I to PLC-γ has itself been shown to be regulated by the phosphorylation of TFII-I on tyrosine residues 357 and 462 by Bruton’s tyrosine kinase (BTK) in B cells (Egloff and Desiderio, 2001). This phosphorylation is proposed to alter the conformational state and allow competitive interaction with PLC-γ, but whether this process is carried out by an equivalent kinase in the brain remains unknown. In addition, Src itself has been shown to be an obligatory participant in TRPC3 channel activation, through an as yet unknown mechanism that does not involve either direct interaction or phosphorylation of TRPC3 (Vazquez et al, 2004).

Altered localization and/or regulation of TRPC3 is an attractive mechanism that could explain many of the features seen in the *Src* mutant mouse as well as in individuals with WBS. For example, TRPC3-mediated calcium currents are activated in the hippocampus in response to the release of activity-dependent growth factors (Li et al, 2010), and the expression profile TRPC3 in the ear suggests it may contribute to development of cochlear tissues and play an important role in hair cell calcium homeostasis and regulation of auditory neurotransmission via G protein-coupled receptor signaling (Phan et al, 2010; Tadros et al, 2010). Investigation of the relationship between TFII-I, TRPC3, and agonist-induced calcium entry was initially established in B cells (Caraveo et al, 2006), but we have confirmed these findings in primary cortical neurons from mice that are heterozygously deleted or duplicated for *Gtf2i* (our unpublished observations). Reduced Src phosphorylation of TFII-I may also impact significantly on its function as a transcriptional activator in the nucleus since phosphorylation-deficient mutants failed to activate the *c-fos* promoter (Cheriyath et al, 2002).

Our results reveal a possible mechanistic link between Src signaling and the dual cellular roles of TFII-I. Recent analysis of a *Gtf2i* heterozygous deficient mouse also identified increased measures of social interaction, but no deficits in growth, craniofacial morphology, motor performance, startle response, or anxiety (Sakurai et al, 2011). Thus, we can conclude that an equivalent reduction of TFII-I activity alone cannot account for the varied phenotypes seen in the *Src* mutant mouse, nor in WBS. Disruption of TFII-I-mediated transcriptional activation and/or TRPC3-mediated calcium transport may, however, still underlie some of the cognitive and behavioral deficits in WBS individuals and *Src* mutant mice. A closely related protein, GTF2IRD1, is also deleted in WBS and has been linked to increased social interaction in mice, as well as deficits in cued fear conditioning, growth delay, hearing loss, and craniofacial abnormalities (Tassabehji et al, 2005; Young et al, 2008; Canales et al, 2014). It is possible that these two proteins both play a role in WBS, perhaps in a combinatorial manner, and that both are regulated by Src and have dual cellular functions. Deletion of the entire WBS syntenic region in mice does recapitulate many of the core features of WBS, including hypersoacibility, craniofacial abnormalities, reduced body weight, increased startle response, and impairments in motor coordination (Segura-Puimedon et al, 2014). Whether these symptoms are due primarily to the deletion of *Gtf2i* and *Gtf2ird1*, or whether additional genes are involved, remains to be determined.

In summary, we have identified a spectrum of neurobehavioral deficits in *Src* mutant mice, some of which overlap with the distinctive cognitive and behavioral profile of individuals with WBS. We have shown that *Src^thl/thl^* mice have altered localization of TFII-I (coded for by a gene commonly deleted in WBS) and the TRPC3 calcium channel, which is regulated by both TFII-I and Src. This study establishes a link between Src signaling and WBS, a disorder with altered social behavior, and opens the possibility of targeting the Src pathway for therapeutic intervention in the future. Additional studies will be needed to determine the role of both Src and TFII-I in neuronal cells, and to understand how their disruption may contribute to neurodevelopmental disorders.

## Synthesis

The decision was a result of the Reviewing Editor Karen Szumlinski and the peer reviewers coming together and discussing their recommendations until a consensus was reached. A fact-based synthesis statement explaining their decision and outlining what is needed to prepare a revision is listed below.

The report, entitled "Disruption of Src is associated with phenotypes related to Williams-Beuren syndrome and altered cellular localization of TFII-I" was deemed acceptable with major revisions to the text, following consultation with 2 reviewers with expertise in kinase neurobiology. Both reviewers felt that the manuscript was generally well-written and agreed that the data presented provided potentially interesting information pertaining to a potential animal model of Williams-Beuren Syndrome (WBS). Reviewer 1, however, questioned the relevance of the behavioral characterization of Src mutant mice for understanding autism spectrum disorders (ASD) as there was little discussion of the data within the context of ASD or the continuum between WBS and ASD. While there was enthusiasm for the Src mutant model of WBS and for the report, in general, both reviewers expressed some very serious concerns regarding specific methodologies and the statistical analyses of the data that negatively impacted their decisions on its suitability for eNeuro. Both reviewers had issue with the lack of procedural details, particularly for the various social interaction tests, and justification of the use of specific procedures in the characterization of the mice. Related to this, Reviewer 1 noted a lack of consistency was noted in the labeling of experimental groups and procedures, which produced confusion for the reader when transitioning from the different subsections of the report. Both reviewers also expressed major concern regarding the superficial statistical approaches to the data and requested that the authors be more explicit in their description of the statistical approaches employed and the results of those statistical approaches. Disagreement in sample sizes/degrees of freedom were also noted across experiments that apparently employed the same animals that require clarification. Low samples sizes for the immunoblotting studies raised concerns regarding statistical power to detect significant group differences. While Reviewer 1 found the Discussion to be fair, it was not thorough nor was it directed objectively at the results of the studies presented therein, particularly within the context of other models of WBS. I concur with the assessments of this report by our expert reviewers and agree that major revisions are required on this report in order to render it more acceptable for publication in eNeuro.

Reviewer Comments:

Reviewer #1:

The current manuscript characterized the behavioral phenotype of Srcthl/thl mutant mice in order to study the neurobiological basis of Williams-Beuren Syndrome (WBS). After a battery of behavioral and molecular experiments, the authors conclude that Src mutants recapitulate several endophenotypes that are expressed in WBS patients. While the results are potentially interesting, several concerns regarding the experimental design, as well as the organization of the manuscript, limit my enthusiasm for the work presented. My specific comments are listed below.

Abstract

1. In the abstract, the authors need to provide the definition for the acronym TRPC3.

Introduction

2. The goals stated in the Introduction seem to be disconnected from the Title of the manuscript. Specifically, the Introduction suggests that the behavioral characterization of the mutant mice resembles Autism spectrum disorder [ASD], as well as WBS, whereas the title emphasizes WBS alone. The manuscript would flow easier if the authors simply focused on WBS, or expand and clarify on the specific manner by which the mutant mice exhibit the continuum between ASD and WBS.

Methods

3. As written, it is not clear if different cohorts of animals were used across all of the different behavioral assays. If the same animals were used, then a time-line depicting the sequence of testing procedures would be helpful to the reader.

4. It is unclear why the authors selected the various social interaction behavioral tasks to characterize WBS sociability (i.e., neutral cage, social novelty, recognition test, and tube test). The authors need to explain the rationale of how each social test individually contributes in the characterization of increased/uninhibited sociability of the WBS phenotype.

5. The description of the "Social interaction test in a neutral cage" is not sufficient for replication purposes. The authors need to operationally define the difference between "Each mouse" (line 125), and the "test" mouse (line 127). It is not clear which animal is the experimental animal (genotype) or the potential social target (non-experimental). Also, was the experimental animal habituated to the new cage initially? If so, for how long? Where was the social target mouse placed in relation to the experimental animal when it was introduced to the novel cage? Lastly, the authors need to operationally define the different dependent variables measured (what was considered cage exploration? Was this variable scored pre- and/or post- the introduction of the social target?).

6. The specific dimensions of the open field and elevated plus maze need to be provided. Also, the authors need to specify what the dimensions of the open field "centre" are, in relation to the outer zone. How long (time) were the animals in the open arena? Similarly, the authors also need to operationally define what is considered time in the open versus closed arms of the elevated plus maze (do the authors include the time spent in the center as time in the open arms or the closed arms? Or is the time spent in the center of the EPM not recorded at all?). How long (time) did the elevated plus maze test last (5 minutes)? Was the mouse placed in the center of the elevated plus maze (facing open or closed arm)? What lighting (lux) was used in the testing room?

7. In the "Ultrasonic Vocalization (USV) subsection of the Methods, the authors need to explicitly describe the "three categories" that they refer to on line 172. Also, what is the reasoning for only habituating the animals to the testing room for 5 minutes prior to testing? Furthermore, it is not clear to me why there are procedural differences between the "male-female" versus the "female-female" social interactions - this section is difficult to follow (lines 144-173). If I understood this correctly, the male-female interactions included wt-mutant pairings, while the female-female interactions did not (i.e., only wt-wt, or mutant-mutant). Would this not preclude the wt-mutant interaction within the female-female paradigm?

8. For the "Balance beam test" why did the authors use "median" values as opposed to averages for data analyses? This is particularly confusing when looking at the data (Figure 5A), which states that the results are reported as a mean plus/minus SEM (see line 1020).

9. The Morris water maze approach is not entirely clear as written. Since there are many variations implemented on this behavioral procedure across the literature (i.e., different number of trials, number of days, intertrial delays, etc). Why did the authors select 2 trials per day, for a total of 6 consecutive days? This approach seems a bit challenging since the demands of the task increase with decreased number of training trials and longer intertrial periods. Also, it is not clear what the authors mean by stating that "the visible platform" was flagged with a small visual cue (line 220). Does this mean that the platform was directly visible "above" the water? Or was the platform submerged under water with a spatial cue over it?

10. The paragraph within line 229-233 seems out of place. It would flow easier if this section was moved to the beginning of the Morris water maze section.

11. A subheading for the "Shock threshold" experiment is needed in the general Methods section (Line 263), as well as in the Results section (line 521).

12. Line 264. The sentence needs to be reformatted.

13. In the "visual-object and novel recognition" section, it is not clear what the authors mean by "having the animals return to explore the object for three continuous 5 min sessions." Does this mean that the animal was removed and placed in the holding cage in between sessions (line 278)? This procedure needs to be clarified (also providing a reference where this task has been previously validated would be helpful).

14. In the "Statistical Analysis" section, the verbiage pertaining to the computer programs used to graph the data is not needed and should be removed (i.e., first sentence of this section; line 366). Also, the description of the statistical analyses themselves needs to be clarified and expanded (lines 367-369). In other words, the authors need to delineate the specific ANOVA techniques used (i.e., 1-way, 2-way, or repeated measures) to assess how genotype influenced the various behavioral responses - simply stating "multiple group data" (line 367) is non-specific. Lastly, line 372 is also unnecessary and should be removed.

Results

15. The order by which the various experimental results (data analyses) are presented in the general Results section does not follow the sequence originally presented in the Methods, making it difficult to follow along. To maintain consistency, the authors should re-arrange the Results and/or Methods section to match one another.

16. Also, the general style by which the statistical analyses are written is vague and creates confusion. The authors need to explicitly identify the main effects and/or interactions per ANOVA, and further provide "direction" (i.e., increase or decrease) to their post-hoc comparisons across the results. For example, simply stating, "There was a significant difference between WT and Src mice in... " (Line 529) makes it very difficult for the reader to understand the effect of genotype on the various dependent variables of the "visual object recognition task" section. Lastly, the authors should clearly delineate how spending more, or less, time with the displaced object influences memory in the Methods section.

17. Also, the authors need to adhere to the guidelines of the journal when reporting F and p values. For example, the actual p value needs to be reported, rather than using the > or < symbol style.

18. Why do the degrees of freedom change across the ANOVAS in the first social interaction experiment (Figure 1A-B)? The 3 different ANOVAs reported across lines 414-417 display different degrees of freedom, even though the authors report an n of 9 per group (i.e., same experiment, with different dependent variables). If individual animals are being removed from specific analyses, the authors should provide justification for doing so. Moreover, the data from this experiment, specifically, needs to be re-analyzed using a 2-way ANOVA with genotype (WT vs mutant) and social target (cup vs novel mouse) as sources of variance. By doing so, the authors can better examine if the main effect for social target (cup vs novel mouse) is dependent on genotype, which in turn would allow the authors to interpret the data as the mutant animals expressing higher levels of social preference at baseline levels. Furthermore, this statistical approach (i.e., 2-way ANOVA) would also uncover if a significant interaction between genotype and social target preference exists, and thus allows the authors to perform all pairwise comparisons.

19. The inclusion of additional asterisks to highlight statistical differences (below .05 alpha values) between the groups is distracting, and thus need to be removed - since the authors specifically indicate in the "Data Analysis" section of the Methods, that the specific alpha level used to determine statistical significance was p < 0.05 (line 371).

20. In line 423, the authors should refrain from using the word "object" since they are referring to Mouse 1 and Mouse 2.

21. The authors should keep consistency on the labels used to describe specific behavioral tests across the sections of the manuscript. For example, in the Results section, the authors use the subheading "Direct social interaction test" (line 426) when describing the data for the "Social interaction test in a neutral cage" (line 124 in the Methods section). Also, in the Methods section, the authors indicate that this behavioral measure was a 5 min test (line 130), whereas in the Results section, they state that this was a 10 min test (line 427). Lastly, the authors report that the mutant mice "spent more time involved in active social approaches than WT mice". This sentence is too vague and non-specific. The authors need to provide the specific dependent variables measured that are displayed in Figure 2C (i.e., sniffing).

22. Under the "Tube test" subsection of the Results, the authors abruptly state "Both genotypes showed dishabituation towards a new smell on trial 7 of an olfactory test (p > 0.05, ANOVA), indicating an intact olfactory system (Figure 2E)." I am not sure if I missed it, but I could not locate the description of this test in the Methods section of the manuscript. Either way, the authors need to clarify how this data was analyzed (i.e., 2-way repeated measures ANOVA?) and report the actual F and p values for the main effects and interaction.

23. In the "Social reunion induced USVs" subsection of the Results, the manner by which the data is presented is not clear. The authors need to clarify that this data was analyzed using a 2-way repeated measures ANOVA with "genotype" and "time" as sources of variance. As written, I believe that the authors suggest that a main effect for genotype was significant (line 445), however, they fail to provide the F value. Furthermore, without a significant interaction (genotype X time), the individual comparisons across the minute-bins are not justified. This trend of unjustified comparisons is also problematic across the data in Figure 3C, 3D, and 3E.

24. The results of the open field are problematic. First, the p<0.005 value presented in line 458 does not clearly indicate if its for a main effect of genotype or for the interaction of genotype and time bin (for the total time of testing; i.e., 30 minutes). Additionally, it looks like the mutant mice are displaying increased general locomotor activity and as such, negates the authors' interpretation on anxiety-like behavior. To make things more complicated, the data from the elevated plus maze further suggests that no differences in sensitivity to anxiogenic stimuli are evident. Did the authors record total locomotor activity in the elevated plus maze? If so, this data would assist in discerning between a general locomotor-induced effect, versus a trait dependent anxiogenic phenotype.

25. In line 470, the authors indicate that the same group of mice were used for both the grip strength and foot slip tests (on line 468), however, the n's for the WT group change from 13 to 16 in Figure 5A-B and 5C-D. Similarly, there is an inconsistency in the number of animals used for the Acoustic startle response test. Figure 6 indicates that there were 12 animals per group, yet, in the Results section (lines 484-485), the degrees of freedom do not reflect this number.

26. The data in Figure 7B for the Morris water maze is difficult to understand. What is an averaged non-target position? This dependent variable was never operationally defined in the Methods section. Furthermore, this data set also needs to be re-analyzed using an independent 2-way ANOVA with genotype and target as sources of variance. Also, the data from Figure 7A should be introduced before the data from 7B, as it is depicted in the Figure.

27. The data analyses of the Fear Conditioning experiment are difficult to follow as it is written in the Results section (and as presented in Figure 7C-D). As is, the horizontal lines in the Figure make it difficult to understand what specific comparisons are being made. Traditionally, a horizontal line over a specific group suggests a significant main effect, however, given the manner that the data is explained in the results section, it becomes clear that this is not the case.

28. The individual statistical analyses (F ratios and p values) for the immunocytochemistry experiment are missing (see lines 547-558).

Discussion

29. The discussion reads well, and the authors make fair observations and interpretations of the data. However, the authors ignore several inconsistencies across their behavioral data. For example, while the mutants had a preference for sniffing a mouse, versus a cup, these mice did not display a preference over a "novel" social target mouse. As such, this finding may indicate that underlying learning/memory aspects may be impaired, rather than general increased social behavior. Although the authors indicate that this may reflect a problem with "habituation" (line 602), it is not clear how habituation, within their experimental approach specifically, is playing a role in such behavioral response.

30. The authors indicate that the social dominance exhibited by the mutant mice is not the result of increased aggression (line 591). Then, the authors lend support to this statement by relying on observations that were not quantified in their experimental design. As such, the veracity of the statement cannot be verified, and thus, should be removed.

31. Given the smaller size (and weight) of the Src mutants, when compared to WT mice, the interpretability and standardization of the behavioral responses across the different assays is weakened - specifically limiting the translational nature of the Src mutant as a model of WBS. This issue needs to be thoroughly addressed in the discussion section, in particular with regards to the behavioral tests that are significantly influenced by differences in weight (such as swimming ability on the water maze, locomotor activity on the open field and rotarod, etc).

32. Overall, the authors need to take a more objective approach to their data and report what they have found, not what they expected to find. As such, the authors should discuss how collectively the behavioral phenotypes observed are similar and/or different from other recent mouse models of WBS (Segura-Puimedon et al., Hum Mol Genet, 2014).

Other Minor Point

33. Keep consistency among your acronyms. In some instances, "hour" is spelled out (line 166), whereas in others, the acronym "h" is used (line 80). Similarly, the description for using NIH-Image (line 96), versus Image J (line 328 and 364) is not consistent. Also, C57BL/6J (line 384), versus C57B6 (line 128). The acronym for "seconds" and "minutes" sometimes is followed by a period (i.e., sec.), while in others it does not (compare lines 228 and 230). Additionally, the authors sometimes use the word "centre" (line 275), while in others, they use the word "center" (line 216). Lastly, it would be helpful to be consistent in the manner by which the authors indicate the age of the animals (in some cases, they state the age in terms of "month" (line 98), while in others they use "weeks" (lines 117 and 175).

34. Lines 284-292 need to be deleted (same paragraph from lines 274-283).

35. The legend for Figure 2E is cut off.

36. In Figure 4A, the equal sign is missing from the Src group on the figure legend.

37. Please make sure that the References are accurate. For example, in the references by Kishenbaum GS et al (PNAS), and Levy D et al (Neuron), all authors need to be listed (unless it exceeds 20 authors, per the journal's guidelines).

38. There is an additional space after the word synchrondrosis in line 942.

Reviewer #2:

In this study authors have characterized mice with a spontaneous null Src mutation for social and cognitive paradigms relevant to neurobehavioral syndromes such as Williams-Beuren syndrome (WBS). They have identified increased social interaction and recognition with deficits in learning and memory. Moreover, they demonstrate altered cellular localization of both TFII-I and TRPC3 in the Src mutants. Based on these results, they conclude that disruption of Src can phenocopy behavioral phenotypes observed in WBS through its regulation of TFII-I.

The manuscript is well written, the experiments seem well performed and the data are generally convincing. Specific comments in relation to this manuscript include:

Did authors use an anesthetic or administer something before mice decapitation?

Regard to procedures for isolation of cytoplasmic and nuclear compartments, it was done without checking the purity of isolated fractions. So, it would be important to check their purity with appropriate marker proteins by western blot and present in Results section.

In the Figure 9A the expression levels of loading control Nucleolin should not vary between the sample lanes.

In the Figure 9B the intensity of bands for Srcthl/thl (western blot) do not match with the values intensity presented in the graph (intensity of these bands has the lower intensity but it is represented in the graph greater intensity compared to WT bands).

For western blot at least 6 samples should be done for statistical analysis.

Western blot band densities and fluorescence intensity should be analyzed using one way ANOVA and present with F and p values.

## Author Response

**Reviewer #1 Comments:**

**Abstract**

1. In the abstract, the authors need to provide the definition for the acronym TRPC3.

**We have included the definition of TRPC3 in the abstract [lines 10-11].**

**Introduction**

2. The goals stated in the Introduction seem to be disconnected from the Title of the manuscript. Specifically, the Introduction suggests that the behavioral characterization of the mutant mice resembles Autism spectrum disorder [ASD], as well as WBS, whereas the title emphasizes WBS alone. The manuscript would flow easier if the authors simply focused on WBS, or expand and clarify on the specific manner by which the mutant mice exhibit the continuum between ASD and WBS.

**We agree that the mention of ASD in the introduction is somewhat confusing. Since we would still like to draw attention to the fact that altering Src function in the opposite direction might be linked with decreased social interaction, either through duplication of GTF2I or through disruption of Src inhibition, we have moved this section to the Discussion [lines 678-687].**

**Methods**

3. As written, it is not clear if different cohorts of animals were used across all of the different behavioral assays. If the same animals were used, then a time-line depicting the sequence of testing procedures would be helpful to the reader.

**We have added a section to the Methods outlining the cohorts used and the order of testing [lines 94-101].**

4. It is unclear why the authors selected the various social interaction behavioral tasks to characterize WBS sociability (i.e., neutral cage, social novelty, recognition test, and tube test). The authors need to explain the rationale of how each social test individually contributes in the characterization of increased/uninhibited sociability of the WBS phenotype.

**We have made suggestions as to how each of the paradigms for testing social interaction may relate to the WBS social phenotype in the Discussion [lines 660-672].**

5. The description of the "Social interaction test in a neutral cage" is not sufficient for replication purposes. The authors need to operationally define the difference between "Each mouse" (line 125), and the "test" mouse (line 127). It is not clear which animal is the experimental animal (genotype) or the potential social target (non-experimental). Also, was the experimental animal habituated to the new cage initially? If so, for how long? Where was the social target mouse placed in relation to the experimental animal when it was introduced to the novel cage? Lastly, the authors need to operationally define the different dependent variables measured (what was considered cage exploration? Was this variable scored pre- and/or post- the introduction of the social target?).

**The procedure for the direct social interaction test has been clarified [lines 125-133].**

6. The specific dimensions of the open field and elevated plus maze need to be provided. Also, the authors need to specify what the dimensions of the open field "centre" are, in relation to the outer zone. How long (time) were the animals in the open arena? Similarly, the authors also need to operationally define what is considered time in the open versus closed arms of the elevated plus maze (do the authors include the time spent in the center as time in the open arms or the closed arms? Or is the time spent in the center of the EPM not recorded at all?). How long (time) did the elevated plus maze test last (5 minutes)? Was the mouse placed in the center of the elevated plus maze (facing open or closed arm)? What lighting (lux) was used in the testing room?

**The procedures for the open field and elevated plus maze tests have been clarified [lines 191-200].**

7. In the "Ultrasonic Vocalization (USV) subsection of the Methods, the authors need to explicitly describe the "three categories" that they refer to on line 172. Also, what is the reasoning for only habituating the animals to the testing room for 5 minutes prior to testing? Furthermore, it is not clear to me why there are procedural differences between the "male-female" versus the "femalefemale" social interactions - this section is difficult to follow (lines 144-173). If I understood this correctly, the male-female interactions included wt-mutant pairings, while the female-female interactions did not (i.e., only wt-wt, or mutant-mutant). Would this not preclude the wt-mutant interaction within the female-female paradigm?

**We have clarified the methods used for the USV testing [lines 169-171]. The three types of calls have now been described in the Methods section and their description referenced [lines 184-185]. WT-mutant female-female interactions were not carried out as all females were housed only with their own genotype. due to competition for food. This has been mentioned in the Methods section [lines 167-169175-177]**

8. For the "Balance beam test" why did the authors use "median" values as opposed to averages for data analyses? This is particularly confusing when looking at the data (Figure 5A), which states that the results are reported as a mean plus/minus SEM (see line 1020).

**We apologise – we did not use the median values but the mean as reported in Figure 5A. We have changed this in the text [line 231].**

9. The Morris water maze approach is not entirely clear as written. Since there are many variations implemented on this behavioral procedure across the literature (i.e., different number of trials, number of days, intertrial delays, etc). Why did the authors select 2 trials per day, for a total of 6 consecutive days? This approach seems a bit challenging since the demands of the task increase with decreased number of training trials and longer intertrial periods. Also, it is not clear what the authors mean by stating that "the visible platform" was flagged with a small visual cue (line 220). Does this mean that the platform was directly visible "above" the water? Or was the platform submerged under water with a spatial cue over it?

**We selected two trials over a six day period to enable us to detect more subtle deficits in visuospatial function – this has been added to the Methods section [lines 244-245]. The platform was submerged with a spatial cue above it – this has been clarified in the text [line 257-258].**

10. The paragraph within line 229-233 seems out of place. It would flow easier if this section was moved to the beginning of the Morris water maze section.

**We have moved this paragraph to the beginning of the Morris water maze section [lines 241-247].**

11. A subheading for the "Shock threshold" experiment is needed in the general Methods section (Line 263), as well as in the Results section (line 521).

**“Shock threshold” subheadings have been added to the Methods and Results sections [lines 297 and 575].**

12. Line 264. The sentence needs to be reformatted.

**This sentence has been reformatted [line 299].**

13. In the "visual-object and novel recognition" section, it is not clear what the authors mean by "having the animals return to explore the object for three continuous 5 min sessions." Does this mean that the animal was removed and placed in the holding cage in between sessions (line 278)? This procedure needs to be clarified (also providing a reference where this task has been previously validated would be helpful).

**The animals were not removed between 5 min sessions, but the three consecutive sessions were analyzed separately. We have added a reference for this procedure, Ng et al, 2012 [lines 303-304].**

14. In the "Statistical Analysis" section, the verbiage pertaining to the computer programs used to graph the data is not needed and should be removed (i.e., first sentence of this section; line 366). Also, the description of the statistical analyses themselves needs to be clarified and expanded (lines 367-369). In other words, the authors need to delineate the specific ANOVA techniques used (i.e., 1- way, 2-way, or repeated measures) to assess how genotype influenced the various behavioral responses - simply stating "multiple group data" (line 367) is non-specific. Lastly, line 372 is also unnecessary and should be removed.

**We have clarified the statistical technique used for each behavioural paradigm [lines 400-403] and unnecessary lines have been removed.**

**Results**

15. The order by which the various experimental results (data analyses) are presented in the general Results section does not follow the sequence originally presented in the Methods, making it difficult to follow along. To maintain consistency, the authors should rearrange the Results and/or Methods section to match one another.

**The Results section has been reordered to match the order in the Methods section.**

16. Also, the general style by which the statistical analyses are written is vague and creates confusion. The authors need to explicitly identify the main effects and/or interactions per ANOVA, and further provide "direction" (i.e., increase or decrease) to their post-hoc comparisons across the results. For example, simply stating, "There was a significant difference between WT and Src mice in... " (Line 529) makes it very difficult for the reader to understand the effect of genotype on the various dependent variables of the "visual object recognition task" section. Lastly, the authors should clearly delineate how spending more, or less, time with the displaced object influences memory in the Methods section.

**We have explicitly identified the main effects found in each behavioural test using the appropriate ANOVA, and provided direction for the post-hoc comparisons throughout the Results section.**

**The statistical analysis of the visual object recognition task has been clarified [lines 585-592].**

**The relationship between time spent exploring displaced or novel objects and memory has been discussed further in the Methods section [lines 324-325 and 332-333].**

17. Also, the authors need to adhere to the guidelines of the journal when reporting F and p values. For example, the actual p value needs to be reported, rather than using the > or < symbol style.

**We have included the actual p values and F values as requested.**

18. Why do the degrees of freedom change across the ANOVAS in the first social interaction experiment (Figure 1A-B)? The 3 different ANOVAs reported across lines 414-417 display different degrees of freedom, even though the authors report an n of 9 per group (i.e., same experiment, with different dependent variables). If individual animals are being removed from specific analyses, the authors should provide justification for doing so. Moreover, the data from this experiment, specifically, needs to be re-analyzed using a 2-way ANOVA with genotype (WT vs mutant) and social target (cup vs novel mouse) as sources of variance. By doing so, the authors can better examine if the main effect for social target (cup vs novel mouse) is dependent on genotype, which in turn would allow the authors to interpret the data as the mutant animals expressing higher levels of social preference at baseline levels. Furthermore, this statistical approach (i.e., 2-way ANOVA) would also uncover if a significant interaction between genotype and social target preference exists, and thus allows the authors to perform all pairwise comparisons.

**The number of mice was n = 9 for the WT group and n = 8 for the mutant group. We have changed the figure legend to reflect this and adjusted the degrees of freedom in the ANOVAs.**

**The data have been re-analyzed using a two-way ANOVA and the results are reported in lines 448-451.**

19. The inclusion of additional asterisks to highlight statistical differences (below .05 alpha values) between the groups is distracting, and thus need to be removed - since the authors specifically indicate in the "Data Analysis" section of the Methods, that the specific alpha level used to determine statistical significance was p < 0.05 (line 371).

**Additional asterisks have been removed from all figures.**

20. In line 423, the authors should refrain from using the word "object" since they are referring to Mouse 1 and Mouse 2.

**We have changed the word object to mouse in this section.**

21. The authors should keep consistency on the labels used to describe specific behavioral tests across the sections of the manuscript. For example, in the Results section, the authors use the subheading "Direct social interaction test" (line 426) when describing the data for the "Social interaction test in a neutral cage" (line 124 in the Methods section). Also, in the Methods section, the authors indicate that this behavioral measure was a 5 min test (line 130), whereas in the Results section, they state that this was a 10 min test (line 427). Lastly, the authors report that the mutant mice "spent more time involved in active social approaches than WT mice". This sentence is too vague and non-specific. The authors need to provide the specific dependent variables measured that are displayed in Figure 2C (i.e., sniffing).

**We have changed the descriptions of the tests to make them consistent between sections. The time for the direct social interaction test was 5 minutes, and this has been changed in the Results section [line 465].**

22. Under the "Tube test" subsection of the Results, the authors abruptly state "Both genotypes showed dishabituation towards a new smell on trial 7 of an olfactory test (p > 0.05, ANOVA), indicating an intact olfactory system (Figure 2E)." I am not sure if I missed it, but I could not locate the description of this test in the Methods section of the manuscript. Either way, the authors need to clarify how this data was analyzed (i.e., 2-way repeated measures ANOVA?) and report the actual F and p values for the main effects and interaction.

**We have included a description of the olfactory testing in the Methods section [lines 141-151], clarified how the data were analyzed and included the statistical values in the Results section [lines 474-476].**

23. In the "Social reunion induced USVs" subsection of the Results, the manner by which the data is presented is not clear. The authors need to clarify that this data was analyzed using a 2-way repeated measures ANOVA with "genotype" and "time" as sources of variance. As written, I believe that the authors suggest that a main effect for genotype was significant (line 445), however, they fail to provide the F value. Furthermore, without a significant interaction (genotype X time), the individual comparisons across the minute-bins are not justified. This trend of unjustified comparisons is also problematic across the data in Figure 3C, 3D, and 3E.

**We have clarified that the data was analyzed using a two-way repeated measures ANOVA, and that there was a significant interaction between genotype and time for both the combined calls and the individual call types (lines 483-499)**

24. The results of the open field are problematic. First, the p<0.005 value presented in line 458 does not clearly indicate if its for a main effect of genotype or for the interaction of genotype and time bin (for the total time of testing; i.e., 30 minutes). Additionally, it looks like the mutant mice are displaying increased general locomotor activity and as such, negates the authors' interpretation on anxiety-like behavior. To make things more complicated, the data from the elevated plus maze further suggests that no differences in sensitivity to anxiogenic stimuli are evident. Did the authors record total locomotor activity in the elevated plus maze? If so, this

data would assist in discerning between a general locomotor-induced effect, versus a trait dependent anxiogenic phenotype.

**We have included the statistical analyses for the effect of genotype and time on the total distance travelled using a two-way ANOVA [lines 504-507]. Total locomotor activity in the elevated plus maze was recorded and there was no effect of genotype – this is now included in the Results [lines 513-514]. This supports our suggestion of a trait dependent anxiogenic phenotype rather than a general locomotor-induced effect in these mice.**

25. In line 470, the authors indicate that the same group of mice were used for both the grip strength and foot slip tests (on line 468), however, the n's for the WT group change from 13 to 16 in Figure 5A-B and 5C-D. Similarly, there is an inconsistency in the number of animals used for the Acoustic startle response test. Figure 6 indicates that there were 12 animals per group, yet, in the Results section (lines 484-485), the degrees of freedom do not reflect this number.

**The same core group of WT mice were used for the grip strength and foot slip tests, but in some cases additional mice were included as part of a demonstration for students, so increasing the WT group number.**

**The number of animals used for the acoustic startle test were reported incorrectly in the Figure. The numbers used were 8 per genotype group and we have corrected Figure 6 to reflect this.**

26. The data in Figure 7B for the Morris water maze is difficult to understand. What is an averaged non-target position? This dependent variable was never operationally defined in the Methods section. Furthermore, this data set also needs to be re-analyzed using an independent 2-way ANOVA with genotype and target as sources of variance. Also, the data from Figure 7A should be introduced before the data from 7B, as it is depicted in the Figure.

**The averaged non-target position refers to the average of the crossings in equivalent areas within each of the three quadrants that do not contain the target platform. We have clarified the text in the Methods [lines 265-267] and Results [lines 548-551] sections.**

**Re-analysis of the data using a two-way ANOVA gave a significant genotype effect (p = .001, F(1,22)=22.32) but no effect of target (p = .3926, F(1,22)=.76). This has been included in the Results section [lines 545-546].**

**The data from Figure 7A has been introduced before that shown in Figure B.**

27. The data analyses of the Fear Conditioning experiment are difficult to follow as it is written in the Results section (and as presented in Figure 7C-D). As is, the horizontal lines in the Figure make it difficult to understand what specific comparisons are being made. Traditionally, a horizontal line over a specific group suggests a significant main effect, however, given the manner that the data is explained in the results section, it becomes clear that this is not the case.

**Figure 7 has been revised so as not to suggest that the asterisks indicate a main group effect.**

28. The individual statistical analyses (F ratios and p values) for the immunocytochemistry experiment are missing (see lines 547- 558).

**The F ratios and p values for the immunocytochemistry analyses have been added.**

**Discussion**

29. The discussion reads well, and the authors make fair observations and interpretations of the data. However, the authors ignore several inconsistencies across their behavioral data. For example, while the mutants had a preference for sniffing a mouse, versus a cup, these mice did not display a preference over a "novel" social target mouse. As such, this finding may indicate that underlying learning/memory aspects may be impaired, rather than general increased social behavior. Although the authors indicate that this may reflect a problem with "habituation" (line 602), it is not clear how habituation, within their experimental approach specifically, is playing a role in such behavioral response.

**We agree that the wording of this section was unclear. We have revised this part of the discussion to include a comparison with the WBS phenotype (as suggested in point #4) and suggest that the lack of novelty recognition with either objects or with mice, may reflect a more general cognitive deficit [lines 668-672].**

30. The authors indicate that the social dominance exhibited by the mutant mice is not the result of increased aggression (line 591). Then, the authors lend support to this statement by relying on observations that were not quantified in their experimental design. As such, the veracity of the statement cannot be verified, and thus, should be removed.

**Since these observations were not quantified, we have changed the statement to reflect the anecdotal nature of the observations [lines 657-658].**

31. Given the smaller size (and weight) of the Src mutants, when compared to WT mice, the interpretability and standardization of the behavioral responses across the different assays is weakened - specifically limiting the translational nature of the Src mutant as a model of WBS. This issue needs to be thoroughly addressed in the discussion section, in particular with regards to the behavioral tests that are significantly influenced by differences in weight (such as swimming ability on the water maze, locomotor activity on the open field and rotarod, etc).

**We are aware that the comparison of mice with significantly different weights/sizes in tests that require motor performance can be problematic. While we cannot completely exclude size effects, we do have confidence in our results because of the following findings. Firstly, in the rotarod test, the mutant and wildtype mice performed equivalently for the first two days, followed by a plateauing of performance in the mutant mice. This suggests that the mice may have a deficit in motor learning and that any deficit in motor performance is mild. Secondly, the escape latencies and swim speed of the mutant mice in the Morris water maze were similar to those of wildtype mice. This suggests that their performance in this test was not due to impaired motor performance or motivation, but was driven by deficit in visuospatial learning and memory. In order to highlight potential problems with the mutant-WT comparison we have added a section to the discussion of the Morris water maze and rotarod [lines 709-718].**

32. Overall, the authors need to take a more objective approach to their data and report what they have found, not what they expected to find. As such, the authors should discuss how collectively the behavioral phenotypes observed are similar and/or different from other recent mouse models of WBS (Segura-Puimedon et al., Hum Mol Genet, 2014).

**We have included a discussion of the phenotypes observed in the recent WBS deletion mouse model, along with those already discussed in the text (*Gtf2i* and *Gtf2ird1* mouse models)[lines 787-792].**

**Other Minor Points.**

33. Keep consistency among your acronyms. In some instances, "hour" is spelled out (line 166), whereas in others, the acronym "h" is used (line 80). Similarly, the description for using NIH-Image (line 96), versus Image J (line 328 and 364) is not consistent. Also, C57BL/6J (line 384), versus C57B6 (line 128). The acronym for "seconds" and "minutes" sometimes is followed by a period (i.e., sec.), while in others it does not (compare lines 228 and 230). Additionally, the authors sometimes use the word "centre" (line 275), while in others, they use the word "center" (line 216). Lastly, it would be helpful to be consistent in the manner by which the authors indicate the age of the animals (in some cases, they state the age in terms of "month" (line 98), while in others they use "weeks" (lines 117 and 175).

**The text has been revised to use consistent language.**

34. Lines 284-292 need to be deleted (same paragraph from lines 274-283).

**These lines have been removed.**

35. The legend for Figure 2E is cut off.

**This has been corrected.**

36. In Figure 4A, the equal sign is missing from the Src group on the figure legend.

**The = sign has been added to Figure 4A**

37. Please make sure that the References are accurate. For example, in the references by Kishenbaum GS et al (PNAS), and Levy D et al (Neuron), all authors need to be listed (unless it exceeds 20 authors, per the journal's guidelines).

**References have been changed to conform to the journal guidelines.**

38. There is an additional space after the word synchrondrosis in line 942.

**The space has been removed.**

**Reviewer #2 Comments:**

1. Did authors use an anesthetic or administer something before mice decapitation?

**All euthanasia was carried out according to approved protocols as stated in the Study Approval section [lines 409-410]. No anaesthetics were used to avoid any unwanted effects on the experimental protocols. For pre-natal pups, this is the method of euthanasia recommended by our Animal Care Committee.**

2. Regard to procedures for isolation of cytoplasmic and nuclear compartments, it was done without checking the purity of isolated fractions. So, it would be important to check their purity with appropriate marker proteins by western blot and present in Results section.

**We understand that the fractionation method does not produce completely pure cellular fractions, which is why we also carried out immunofluorescence to validate the findings from Western blot analysis.**

3. In the Figure 9A the expression levels of loading control Nucleolin should not vary between the sample lanes.

**We understand that the intensity of the nucleolin bands is not uniform across all the sample lanes, but the intensity of the GTF2I signal is adjusted based on the nucleolin band intensity so we believe that the estimate of protein expression is still reliable.**

4. In the Figure 9B the intensity of bands for Srcthl/thl (western blot) do not match with the values intensity presented in the graph (intensity of these bands has the lower intensity but it is represented in the graph greater intensity compared to WT bands). For western blot at least 6 samples should be done for statistical analysis.

**We are not sure what the reviewer is referring to here. The TRPC3 bands in the membrane fraction of the Src^thl/thl^ samples shown in Figure 9B are clearly more intense than those in the WT samples (with fairly uniform N-cadherin control bands) and this is reflected in the graph shown in Figure 9C. We agree that a sample size of six would be preferable, but since we confirmed the findings using an independent method (immunofluorescence) we feel that the samples sizes used are sufficient.**

5. Western blot band densities and fluorescence intensity should be analyzed using one way ANOVA and present with F and p values.

**Since a one way ANOVA is generally used to test if there are any significant differences between the means of three or more independent groups, and we have only two independent groups, we believe that a Student’s t-test is sufficient in this case.**
